# The IL-33-PIN1-IRAK-M axis is critical for type 2 immunity in IL-33-induced allergic airway inflammation

**DOI:** 10.1038/s41467-018-03886-6

**Published:** 2018-04-23

**Authors:** Morris Nechama, Jeahoo Kwon, Shuo Wei, Adrian Tun-Kyi, Robert S. Welner, Iddo Z. Ben-Dov, Mohamed S. Arredouani, John M. Asara, Chun-Hau Chen, Cheng-Yu Tsai, Kyle F. Nelson, Koichi S Kobayashi, Elliot Israel, Xiao Zhen Zhou, Linda K. Nicholson, Kun Ping Lu

**Affiliations:** 1 Division of Translational Therapeutics, Department of Medicine and Cancer Research Institute, Beth Israel Deaconess Medical Center, Harvard Medical School, Boston, MA 02215 USA; 2 Department of Molecular Biology & Genetics, Cornell University, Ithaca, NY 14853 USA; 3 Department of Nephrology and Hypertension, Hadassah-Hebrew Medical Center, 91120 Jerusalem, Israel; 4 Department of Medicine, Brigham and Women’s Hospital, Boston, MA 02115 USA; 5 Department of Microbial Pathogenesis & Immunology, Texas A&M Health Science Center, College Station, TX, 77843 USA; 6 Broad Institute of MIT and Harvard, Cambridge, MA 02142 USA; 7 Institute for Translational Medicine, Fujian Key Laboratory for Translational Research in Cancer and Neurodegenerative Diseases, Fujian Medical University, Fuzhou, 350108 Fujian China

**Keywords:** Antibodies, Inflammatory diseases, Interleukins, Chronic inflammation

## Abstract

Interleukin 33 (IL-33) is among the earliest-released cytokines in response to allergens that orchestrate type 2 immunity. The prolyl *cis-trans* isomerase PIN1 is known to induce cytokines for eosinophil survival and activation by stabilizing cytokines mRNAs, but the function of PIN1 in upstream signaling pathways in asthma is unknown. Here we show that interleukin receptor associated kinase M (IRAK-M) is a PIN1 target critical for IL-33 signaling in allergic asthma. NMR analysis and docking simulations suggest that PIN1 might regulate IRAK-M conformation and function in IL-33 signaling. Upon IL-33-induced airway inflammation, PIN1 is activated for binding with and isomerization of IRAK-M, resulting in IRAK-M nuclear translocation and induction of selected proinflammatory genes in dendritic cells. Thus, the IL-33-PIN1-IRAK-M is an axis critical for dendritic cell activation, type 2 immunity and IL-33 induced airway inflammation.

## Abstract (web summary)

IL-33 orchestrates type 2 immunity in allergic asthma. Here the authors show, using biochemical, structural and patient data, that upon IL-33 or allergic challenge, the isomerase Pin1 modifies IRAK-M to control the production of pro-inflammatory cytokines in the setting of airway inflammation.

## Introduction

Allergic asthma is a T helper type 2 (T_H_2 type) immune disease, characterized by pulmonary infiltration of specific T helper cells^1,2^, and increased secretion of the type 2 cytokines IL-4, -5 and -13. These cytokines are necessary for adaptive T_H_2 immunity development, IgG class switching, goblet cell metaplasia and airway eosinophilia, all of which are hallmarks of allergic asthma^3–5^. The production of type 2 cytokines is regulated by upstream signaling pathways, including those initiated by the Toll-like receptor/interleukin-1 receptor (TLR/IL-1R) superfamily^6,7^. Notably, TLR4, predominantly expressed on pulmonary epithelial cells and alveolar macrophages^8^, mediates asthma induction by Derp2, a major allergen of the house dust mite (HDM)^9^. The activation of such TLRs by airborne allergens often induces T_H_2 response^10^ via mediators such as interleukin 33 (IL-33), a ligand for IL-1R^8,11,12^.

A central function of IL-33 in the pathogenesis of asthma has been reported^12,13^. In the lung, IL-33 comes mainly from epithelial cells that form the first line of defense against inhaled allergens and microorganisms. Upon stimulation, or as a result of tissue damage, IL-33 is secreted to act as both a chemo attractant and an immune modulator to activate cells of both the innate and adaptive arms of immunity^14^. For example, IL-33 1) activates resident dendritic cells (DC) to induce their maturation critical for allergic airway inflammation^15,16^ via DC-stimulated differentiation of T cells into T_H_2 cells (T_H_2 polarization)^17^, 2) activates DCs to promote naive CD4^+^ T cells to produce IL-5 and IL-13^8^, and 3) prolongs human eosinophil survival, adhesion and degranulation^18^ with impacts on both mast cells (to prolong their survival and adhesion and stimulate their cytokine production^19^), and on alveolar macrophages (to stimulate their secretion of IL-13). How these IL-33 signaling pathways are regulated, especially after engaging a TLR/IL-1R, is not fully understood.

A hallmark regulatory mechanism in many signaling pathways including TLR/IL-1R signaling is proline-directed phosphorylation (pSer/Thr-Pro)^20^. Proline uniquely adopts *cis* and *trans* peptide bond conformations, and its isomerization is catalyzed by peptidyl-prolyl *cis-trans* isomerases (PPIases), including PIN1 that is uniquely specific for pSer/Thr-Pro motifs^21,22^. Such conformational exchange can have profound effects on key regulators in many cellular processes^21,22^. Consequently, PIN1 deregulation contributes to the pathogenesis of numerous diseases^22,23^. For example, we have previously shown that PIN1 is a regulator of interleukin receptor associated kinase 1 (IRAK1) activation in TLR signaling^24^. Of specific relevance to asthma are studies showing that PIN1 is abnormally activated in eosinophils in asthmatic airways and that PIN1 increases key cytokine production necessary for eosinophils survival and activation by stabilizing their mRNA half-life^25–28^. However, many questions remain regarding the role of PIN1 regulation of TLR/IL-1R upstream signaling pathways in allergic airway inflammation.

Interleukin receptor associated kinase M (IRAK-M) belongs to the Interleukin receptor associated kinase (IRAK) family whose members share structural domains and participate in signal transduction mediated by TLR/IL-1R^29,30^. IRAK-M is traditionally considered as a negative regulator of TLR/IL-1R signaling by trapping IRAK1 in the activated receptor complex and preventing downstream signaling^31,32^. IRAK-M was also shown to be associated with the pathogenesis of early-onset persistent asthma^33^, but neither its function nor regulation in this process has been investigated.

Here we identify IRAK-M as a PIN1 target upon IL-33 challenge. Our NMR analysis and docking simulations suggest a model for how IRAK-M and PIN1 might regulate IRAK1-mediated pro-inflammatory signaling. To test this model, and to more broadly probe the role of IRAK-M and PIN1 in allergic asthma, IL-33 is used to induce type 2 immunity signaling in a combination of cell lines, mouse models, and primary cells from mouse models. Extension of the relevance of these findings to allergic asthma in humans is suggested by correlation with analyses of samples from human asthmatic participants. Together, our results from complementary approaches that span from atomic to organism levels reveal critical roles of IRAK-M and PIN1 in IL-33-induced type 2 immunity. These results consistently indicate that upon IL-33-induced inflammation, PIN1 is activated, which in turn binds to and catalyzes cis-trans isomerization of phosphorylated IRAK-M, inducing IRAK-M stabilization and nuclear translocation and concomitant expression of a set of pro-inflammatory genes in DCs. This demonstrate that IRAK-M and its regulator PIN1, along with downstream cytokines, control T_H_2 response upon IL-33 challenge. These findings reveal an important axis in type 2 immunity and offer new therapeutic targets for allergic asthma.

## Results

### PIN1 is required for IL-33 induction of allergic asthma

**Fig. 1:**
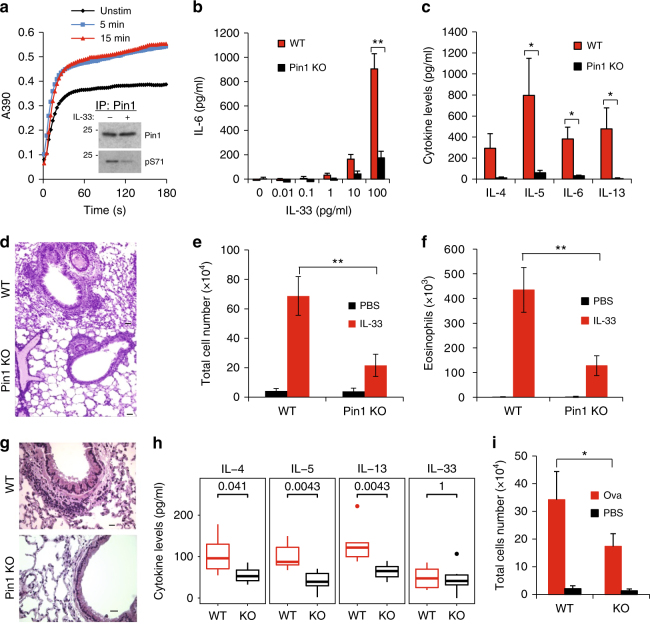
PIN1 is required for IL-33 to induces T_H_2 polarization and allergic inflammation. **a** 5 and15 min after IL-33 treatment of DC2.4 cells, cells were extracted and PIN1 activity was measured as previously described. Inset: PIN1 was immune precipitated and immune blotted for PIN1 or Ser71 phosphorylation as indicated. **b** WT and *PIN1* KO MEF’s were treated with different concentrations of IL-33 for 24 h and the medium was measured for IL-6 secretion. **c** WT and *PIN1* KO mice were treated with 200 ng/mice/day for 4 consecutive days and the BALF was examined for IL-4, 5, 6 and 13 by ELISA. **d** H&E staining representative of lungs from WT and *PIN1* KO mice treated with IL-33 (*n* = 4). Scale bar = 50 µm. **e** Total cell number in the BALF of WT and *PIN1* KO mice was measured using HEMAVET multispecies hematology analyzer. **f** Eosinophil cell number in the BALF of WT and *PIN1* KO mice was measured using HEMAVET multispecies hematology analyzer. **g** Representative H&E staining of lung sections from WT and *PIN1* KO mice after OVA-induced allergic asthma (*n* = 4). Scale bar = 50 µm. **h** IL-4, -5, -13 and IL-33 levels in the BALF of WT and *PIN1* KO mice after OVA-induced allergic asthma. The groups were compared using Mann-Whitney *U* test (**i**) Total cell number in the BALF of WT and *PIN1* KO mice before and after OVA-induced allergic asthma. The data were analyzed by a Student’s two-tailed *t* test unless stated otherwise, and the values are reported as mean ± standard errors of the means (SEM). *- statistical significance (*P* < 0.05), **- significance (*P* < 0.01)

PIN1 enzymatic activity is highly regulated in response to external stimuli^22^. To examine whether IL-33 signaling affects PIN1 enzymatic activity, the dendritic cell line DC2.4 was treated with IL-33, followed by PIN1 enzymatic activity assay (Fig. 1a). Brief (5 min) stimulation with IL-33 dramatically elevated PIN1 activity, which correlated with PIN1 dephosphorylation at Ser 71 (Fig. 1a). PIN1 Phosphorylation at Ser 71 is known to inhibit PIN1 isomerase activity and cellular function^34^.

To assess PIN1 involvement in IL-33 signaling, mouse embryonic fibroblasts (MEFs) derived from PIN1 wild-type (*PIN1* + / + , WT) or knockout (*PIN1*-/-, KO) mice were treated with IL-33, and IL-6 production was measured as a proxy for induction of inflammation (Fig. 1b). *PIN1* KO abolished IL-6 secretion in response to all IL-33 concentrations tested. For in vivo assessment of PIN1 involvement in IL-33 signaling, WT and *PIN1* KO mice were intranasally challenged with IL-33 for 4 days before bronchial alveolar lavage fluids (BALF) were examined for cytokines and cell content (Fig. 1c). After IL-33 challenge, levels of T_H_2 cytokines (IL-4, -5, -6 and -13) in *PIN1* KO mice BALF were drastically lower than those in WT mice. While IL-33-challenged WT mice exhibited moderate to severe inflammation and high levels of infiltrating cells, especially eosinophils, *PIN1* KO mice showed very mild responses to IL-33 with few inflammatory infiltrates and markedly reduced cell counts (Fig. 1d-f). Thus, PIN1 is a crucial factor in IL-33-induced lung inflammation.

To test whether PIN1 involvement is restricted to the IL-33/IL-1R pathway or extends to other allergy-inducing pathways, we evaluated the above parameters in an ovalbumin (OVA) induced model of allergic asthma^35^. Similarly, OVA-challenged *PIN1* KO mice showed reduced lung inflammation (Fig. 1g), reduced T_H_2 cytokine levels in BALF (Fig. 1h) and reduced BALF total cells count (Fig. 1i) compared to challenged WT mice. Thus, PIN1 involvement is not specific to IL-33, and suggests a broad role of PIN1 in allergic asthma

### PIN1 binds to pS110-Pro in IRAK-M upon IL-33 challenge

**Fig. 2:**
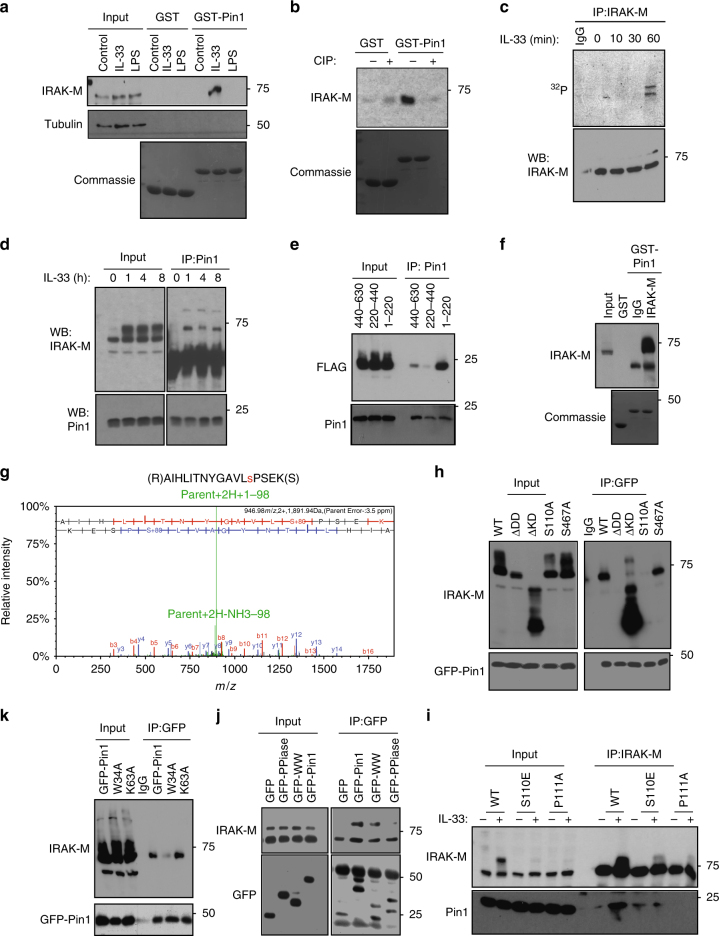
IL-33 induces IRAK-M phosphorylation and binding to PIN1. **a** GST PIN1 pulldown assay with DC2.4 cell extracts either non-treated or treated with IL-33 (100 ng/ml) or LPS (100ng/ml) for 1 h. The GST-PIN1 bound proteins were eluted using reduced gluthatione and probed for IRAK-M. In the lower panel Coomassie blue staining of the blot shows equal amounts of GST or GST-PIN1 that were used for pull-down. **b** GST-PIN1 pulldown of DC2.4 cell extracts either treated or not with IL-33, followed by treatment in the absence or presence of calf intestinal alkaline phosphatase (CIP) for 30 min at room temperature before subjecting to GST-PIN1 pulldown. **c** DC2.4 cells were labeled with 10 μCi/ml {γ-^32^ P}ATP for 3 h. The cells were washed with fresh medium and treated with 100 ng/ml IL-33 for the indicated times prior to IRAK-M immunoprecipitation. **d** DC2.4 cells stably expressing IRAK-M were treated with IL-33 and at the indicated time points were subjected to CO-IP using anti-PIN1 antibody and blotted for IRAK-M. **e** HEK293 cells were transfected with different IRAK-M constructs expressing the N’ terminal domain (aa1-220), the middle portion of the protein (aa220-440) or the C’ terminal domain (aa 440-630), and then treated with IL-33, followed by CO-IP for PIN1. **f** DC2.4 cells stably expressing IRAK-M were treated with IL-33 and subjected to GST or GST-PIN1 pull-down. The bound proteins were eluted and subjected to IP using IRAK-M antibody. **g** LC-MS/MS analysis shows phosphorylation of IRAK-M at position Ser110. **h** WT IRAK-M or its mutants lacking the death domain (IRAK-M ∆DD), lacking the kinase domain (IRAK-M ∆KD), IRAK-M S110A or IRAK-M S467A (where these serine residues were mutated to alanine) were expressed in HEK293 cells, followed by CO-IP for GFP after IL-33 treatment. **i** IRAK-M, S110E or P111A stably expressing DC2.4 cells were stimulated with IL-33 and PIN1 interaction was monitored by CO-IP experiment as indicated. **j** HEK293 cells were co-expressed with IRAK-M and GFP, GFP-PIN1, GFP-WW domain or GFP-PPIase domain and, then treated with IL-33 before CO-IP for GFP. **k** HEK293 cells were co-expressed with IRAK-M and WT PIN1, PIN1 mutant W34A, or PIN1 mutant K63A and then were treated with IL-33 before CO-IP for GFP

Dendritic cells are among the predominant cell types that react to IL-33 stimulation, and are essential for IL-33-dependent allergic inflammation^15,16^. To identify IL-33-dependent PIN1 targets, the dendritic cell line DC 2.4 was treated with IL-33 or LPS for 1 h before GST-PIN1 pull-down assay to identify PIN1-binding proteins^36,37^. We identified specific interaction between PIN1 and IRAK-M after stimulation by IL-33, although not LPS (Fig. 2a). Our interest in IRAK-M stemmed from our prior findings that PIN1 regulates IRAK1 activity in TLR signaling^24^ and IL-33 activates the IRAK1-dependent pathway^38^. As expected, this PIN1-IRAK-M interaction was phosphorylation-dependent (Fig. 2b). Moreover, IRAK-M was phosphorylated in DC2.4 cells at 1 h after IL-33 treatment as detected by [γ-^32^P] in vivo labeling (Fig. 2c), and the PIN1-IRAK-M interaction was evident only after IL-33 treatment, as detected by PIN1co-immunoprecipitation (CO-IP) (Fig. 2d). Thus, PIN1-IRAK-M interaction is evident after IRAK-M phosphorylation following IL-33 stimulation.

To identify the region(s) in IRAK-M responsible for binding PIN1, its N-terminal (aa 1-220), middle (aa 220–440) or C-terminal domain (aa 440–630) were co-expressed in HEK293 cells with the IL-33 receptor ST2^39^. Cells were treated with IL-33 and subjected to CO-IP. PIN1 interaction was mainly evident with IRAK-M N-terminal domain (Fig. 2e, Supplementary Fig 1a). To directly identify IRAK-M phosphorylation site(s) required for PIN1 binding, DC2.4 cells stably overexpressing IRAK-M were treated with IL-33 before GST-PIN1 pull-down. PIN1-bound proteins were then eluted and subjected to IRAK-M IP (Fig. 2f). This IRAK-M was subjected to SDS-PAGE, followed by mass spectrometry, identifying a major phosphorylation site at Ser110 (Fig. 2g), which is followed by a Proline, making it a potential PIN1 binding site^22^.

We next overexpressed in HEK293 cells IRAK-M truncated forms lacking the death domain (IRAK-M ∆DD-) or the kinase domain (IRAK-M ∆KD-), or IRAK-M mutants S110A or IRAK-M S467A, where the suspected Ser (S110 or S467) was substituted to Ala to eliminate potential phosphorylation. The S110A mutation abolished the PIN1-IRAK-M interaction (Fig. 2h, Supplementary Fig 1a). Importantly, absence of the death domain (DD) also abolished the interaction, which suggests that IRAK-M interaction with its protein kinase involves the IRAK-M DD. These pull-down studies identify S110 as the IRAK-M site of interaction with PIN1.

To further confirm S110 in IRAK-M as a pSer-Pro motif for PIN1 interaction, we generated IRAK-M mutants S110E (a phosphomimetic mutant), or P111A (eliminates the PIN1 consensus motif). DC2.4 cells stably expressing IRAK-M, S110E or P111A were treated with IL-33 and subjected to CO-IP experiments. PIN1-IRAK-M interaction was evident only after IL-33 stimulation (Fig. 2i, Supplementary Fig 1a). However, with IRAK-M S110E, much weaker PIN1 interaction was evident before IL-33 stimulation. The P111A mutation totally abolished IRAK-M phosphorylation and PIN1 interaction. These results support S110-P111 in IRAK-M serving as a pSer-Pro interaction site.

To identify which PIN1 domain mediates this interaction, HEK293 cells expressing ST2 were co-overexpressed with IRAK-M and GFP, GFP-PIN1, -WW domain or –PPIase domain. Cells were treated with IL-33, then were subjected to CO-IP for GFP. The PIN1-IRAK-M interaction was based on the PIN1-WW domain and not the PPIase domain (Fig. 2j, Supplementary Fig 1b). CO-IP experiments using WW domain mutant W34A (binding defective) or PPIase domain mutant K63A (catalytically inactive) confirmed PIN1requirement of a functional WW domain (Fig. 2k, Supplementary Fig 1b). Thus, PIN1 binds to the pS110-P motif in IRAK-M via its WW domain upon IL-33 stimulation.

### PIN1 isomerizes the pS110-P motif in IRAK-M

**Fig. 3:**
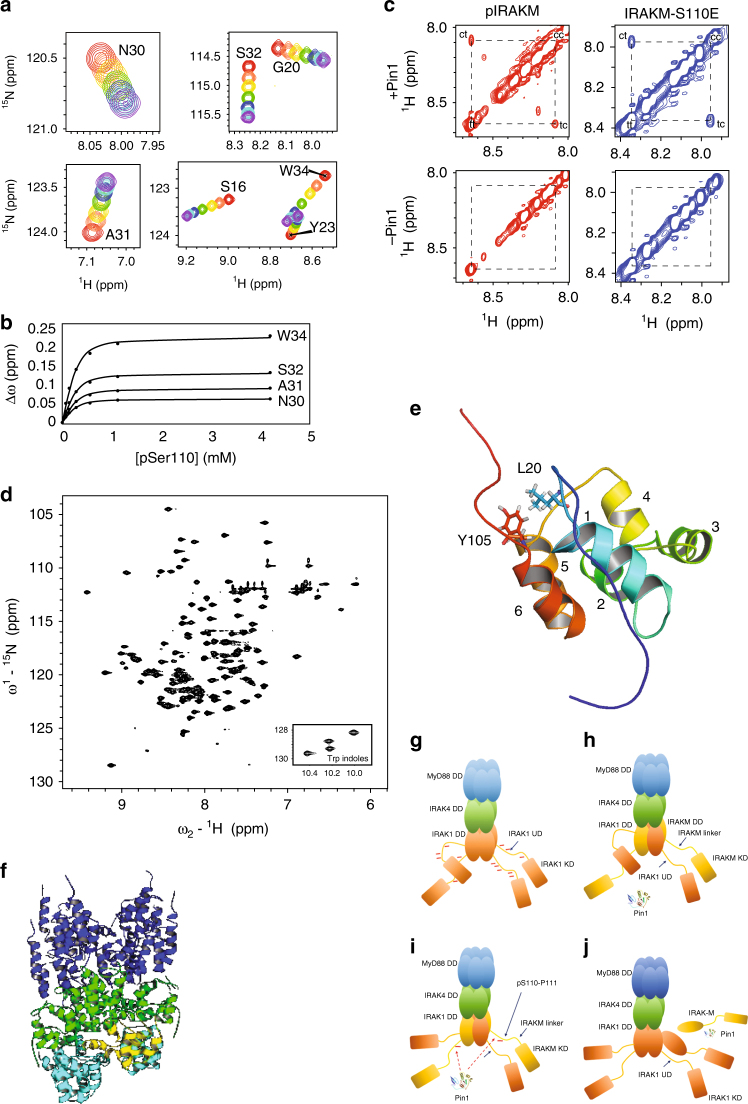
PIN1 binds to and isomerizes the pS110-P111 motif, located C-terminal to the IRAK-M DD. **a** Binding of the PIN1 WW domain to IRAK-M-pS110 peptide is demonstrated by overlaid regions extracted from ^1^H-^15^N HSQC spectra of ^15^N-labeled PIN1 WW domain that show progressive peak shifts with increasing peptide concentration (apo = red, purple = highest concentration). **b** Changes in chemical shift (∆ω) with increasing IRAK-M-pS110 peptide concentration (filled circles) were fit to a simple bimolecular interaction model (solid lines) to yield the apparent dissociation constant, *K*_D_^App^ = 60.7 ± 11.5 µM (mean ± s.d.). **c** PIN1 catalysis of IRAK-M-pS110 and IRAK-M-S110E peptides is demonstrated by ROESY spectra of each peptide in the presence or absence of PIN1. In the presence of PIN1 ( + PIN1, top two spectra), cross peaks between *cis* and *trans* appear for both IRAK-M-pS110 and IRAK-M-S110E peptides. In the absence of PIN1 (-PIN1, bottom two spectra), no cross peaks were observed for either peptide. **d** ^1^H-^15^N HSQC spectrum of cleaved ^15^N-IRAK-M[1–119:R56D,Y61E]. **e** Ribbon diagram of the IRAK-M DD structure determined by NMR, showing the six alpha helices and highlighting the Y105-L20 interaction that anchors the C-terminal tail to the structure. **f** A ClusPro generated model of IRAK-M DD docked into a modified Myddosome oligomer containing three IRAK1 DD subunits in the L, M and N positions in the 3MOP structure. Blue: six MyD88 DD subunits, Green: Four IRAK4 DD subunits, Cyan: three IRAK1 DD homology model subunits, Yellow: docked NMR structure of the IRAK-M DD. **g**–**j** Proposed model for IRAK-M and PIN1 regulation of IRAK1 mediated immunity signaling. **g** IRAK1 homotetramer assembled on the Myddosome is able to achieve full hyperphosphorylation through each IRAK1 undefined domain (UD) being phosphorylated by neighboring IRAK1 kinase domains (KDs). **h** In the presence of IRAK-M, heterotetrameric IRAK-M/IRAK1 assembles on the Myddosome where IRAK-M DD replaces every-other IRAK1 DD subunit, preventing further IRAK1 hyper-phosphorylation and inhibiting IRAK1 release from the Myddosome thereby suppressing IRAK1-mediated inflammation. **i** IRAK1 KD phosphorylates S110 in next-neighbor IRAK-M. **j** Binding of PIN1 to IRAK-M pS110P (IRAK-M phosphorylated at residue S110) and subsequent isomerization induces release of IRAK-M from the Myddosome for downstream PIN1- and IRAK-M dependent signaling

To directly observe PIN1 binding and isomerization of the IRAK-M pS110-P motif, we used nuclear magnetic resonance (NMR). In the two-dimensional (2D) ^1^H-^15^N Heteronuclear single quantum coherence (HSQC) spectrum of a ^15^N-labeled protein, each backbone NH produces a peak at a specific position that reflects its average chemical environment. Peaks in this spectrum thereby serve as sensors to detect and quantify ligand binding. When ^15^N-PIN1-WW was titrated with unlabeled IRAK-M phosphopeptide (IRAK-M-pS110) or the corresponding phosphomimetic mutant IRAK-M-S110E, several peaks in the ^1^H-^15^N HSQC spectrum moved with increasing peptide concentration (Fig. 3a, Supplementary Fig 2a). This peak movement (∆δ) demonstrated fast binding kinetics, and allowed the corresponding dissociation constant (*K*_D_) for each peptide to be determined (Fig. 3b).

To investigate PIN1-catalyzed isomerization of the IRAK-M-pS110 and IRAK-M-S110E peptides, we performed 2D rotating-frame Overhauser effect spectroscopy (ROESY) NMR experiments. When PIN1 was present, exchange cross-peaks between *cis* and *trans* isomers appeared in ROESY spectra of both IRAK-M-pS110 and IRAK-M-S110E peptides, but were not observed when PIN1 was absent (Fig. 3c). Quantitative analysis of the ROESY data yielded PIN1-catalyzed isomerization rates for both peptides (Table 1). These results indicate that PIN1 not only binds to, but also catalyzes the *cis/trans* isomerization of the pS110-P motif and its S110E phosphomimetic mutant in IRAK-M.

**Table 1:** Pin1-catalyzed isomerization rates of IRAK-M-pS110 and IRAK-M-S110E peptides

	pIRAK-M-pS110	IRAK-M-S110E
Peptide concentration	4.4 mM	4.18 mM
PIN1 concentration	13.8 µM	20 µM
*trans/cis* ratio (*K*_isom_)	10.7	6.87
*k*_ex_ (s^−1^)	28.98	0.84
*k*_tc_ (s^−1^)	2.48	0.11
*k*_ct_ (s^−1^)	26.50	0.73

### IRAK-M DD structure and docking suggest regulatory model

To locate the pS110-P111 motif relative to the IRAK-M DD fold and to gain mechanistic insight regarding PIN1 binding and isomerization of this motif, we attempted to express and purify recombinant IRAK-M residues 1–119 (IRAK-M[1–119]), including the DD and part of the linker between the death and kinase domains. Although a fusion protein containing the maltose binding protein (MBP) and IRAK-M[1–119] was soluble, the cleaved IRAK-M[1–119] precipitated under all conditions explored. To inhibit possible surface charge-mediated precipitation, two residues predicted to be solvent exposed in the monomer, R56 and Y61, were mutated to negatively charged residues (D and E, respectively) to impart electrostatic repulsion. These mutations yielded a soluble cleaved IRAK-M [1–119:R56D,Y61E] monomer, ascertained by size exclusion chromatography.

The ^1^H-^15^N HSQC spectrum of ^15^N-IRAK-M [1–119:R56D,Y61E] shows well-dispersed peaks, indicating that the domain is folded (Fig. 3d). The backbone (97% complete) and sidechain (85% complete) ^13^C, ^15^N and ^1^H assignments were determined for IRAK-M [1–119:R56D,Y61E] and are deposited at the BioMagResBank (http://www.bmrb.wisc.edu/, accession number 30237). This folded monomeric IRAK-M [1–119:R56D,Y61E] mutant was used for NMR structure determination.

Standard three-dimensional NOESY experiments along with chemical shift constraints yielded the high-resolution NMR structure of IRAK-M [1-119:R56D,Y61E] (PDB ID 5UKE). The structure adopts the canonical DD fold, demonstrating that the R56D and Y61E mutations do not disrupt the native fold. This structure consists of six α-helices: α1 (P22 – S34), α2 (W41 – L48), α3 (D54 – V62), α4 (T69 – S75), α5 (I83 – M93) and α6 (R96 – N104) (Fig. 3e). The ensemble of lowest energy structures shows a well defined α-helical core (residues 14-47, 56-105) with a backbone RMSD of 0.19 Å. The overall structure quality was slightly better than the average NMR structure in terms of clash score (11) and sidechain outliers (9%), and slightly worse in Ramachandran outliers (3%). Although the N- and C-terminal regions (M1 – P21 and Y105 – G124, respectively) do not adopt regular secondary structure, residues A10 to P21 form a loop that packs against residues in α1, α6, the α4-α5 loop, and Y105 at the beginning of the C-terminal tail (Fig. 3e). The Y105 aromatic ring associates with L20 and P21 sidechains, stabilizing this N- to C-terminal tail interaction. The S110-P111 motif is solvent-exposed in the downstream unstructured part of the C-terminal tail, suggesting accessibility for interactions with kinase(s), phosphatase(s), and PIN1.

It was previously reported that IRAK-M reduces IRAK1 phosphorylation, increases IRAK1 association with MyD88, and inhibits formation of the downstream IRAK1-TRAF signaling complex^31^. The structural mechanism by which these effects are mediated by IRAK-M are unknown. To investigate possible interactions that might facilitate these functiones of IRAK-M in IRAK1-mediated signaling, docking simulations were performed using a Myddosome-based model containing IRAK1 DD in place of IRAK2 DD subunits using the program ClusPro^40–43^. IRAK-M was docked with a modified Myddosome composed of six MyD88, four IRAK4, three IRAK1 DD subunits, and an empty IRAK1 DD subunit space. For each of the four scoring schemes applied, the resulting ten models (representing highly populated clusters of low energy complexes) included IRAK-M DD docked into the empty subunit position in the canonical orientation. These docked models revealed favorable IRAK-M DD interactions with both IRAK1 DD and IRAK4 DD (Fig. 3f, Supplementary Fig. 2b,c).

For comparison, IRAK-M was also docked with a Myddosome complex composed of six MyD88, four IRAK4, three IRAK2 DD subunits and an empty IRAK2 DD subunit space. In contrast to the above findings for IRAK-M docking into an IRAK1-containing Myddosome, these simulations for an IRAK2-containing Myddosome yielded no models with IRAK-M docked into the empty subunit position in the canonical orientation. Repulsion between IRAK-M E51 and IRAK2 D14 and absence of a favorable IRAK-M D38 interaction with IRAK1 R61 (S47 in IRAK2) could explain this result. This comparison suggests distinct mechanisms for interaction of IRAK-M with the Myddosome, depending on whether it contains IRAK2 or IRAK1.

These docking simulations suggest that the Myddosome framework allows formation of an IRAK1-IRAK-M hetero-tetramer and suggest a model by which IRAK1 can phosphorylate IRAK-M S110P upon stimulation (Fig. 3g-j). In this proposed model, upon stimulation IRAK1 assembles onto the Myddosome and becomes hyper-phosphorylated (Fig. 3g). Our docking simulations suggest that the IRAK-M DD might replace every other IRAK1 DD subunit in the Myddosome complex, thereby forming a heterotetramer with IRAK1 (Fig. 3h). This alternating IRAK1/IRAK-M structure might prevent release of hyper-phosphorylated IRAK1 from the Myddosome, inhibit further IRAK1 molecules from hyper-phosphorylation, and suppress IRAK1-mediated inflammation (Fig. 3i). PIN1 binding to IRAK-M pS110P and subsequent isomerization could displace the C-terminal region of IRAK-M DD, thereby disrupting the Y105-mediated N-to-C interaction and inducing IRAK-M dissociation from the Myddosome (Fig. 3j) for downstream IRAK-M dependent signaling.

This proposed model, based on our NMR structure and subsequent docking simulations, inspires three key hypotheses: 1) formation of an IRAK1-IRAK-M heterotetramer on the Myddosome could prevent IRAK1 activation; 2) IRAK1 could phoshorylate IRAK-M on S110P, generating a potential PIN1 recognition site; and 3) PIN1 binding and isomerization of the pS110-P motif in IRAK-M could promote IRAK-M release from Myddosome complex, inducing IRAK-M dependent signaling. Hypothesis 1 could explain the known effects of IRAK-M on IRAK1 phosphorylation, signaling and association with MyD88^31^. To further test hypotheses 2 and 3, experiments were performed to determine whether IRAK-M is phosphorylated by IRAK1, and to investigate the role of PIN1 and pS110-P in IRAK-M-dependent signaling.

### PIN1 promotes IRAK-M nuclear translocation and stability

The above model suggests that IRAK1 might phosphorylate IRAK-M, which could then recruit PIN1 to induce IRAK-M conformational change, causing release of IRAK-M from the Myddosome. An IRAK-M mobility shift in SDS-PAGE gels in IRAK1 wild-type 293 cells but not in IRAK1-null 293 cells suggests that IRAK-M is phosphorylated in an IRAK1-dependent manner (Supplementary Fig 2d). Moreover, DCs stimulated with IL-33 showed IRAK1 activation as indicated by a mobility shift (Supplementary Fig 2e). Subsequent IP of IRAK1 and incubation with either purified GST-IRAK-M containing amino acids 1-120 or its S110A mutant in the presence of γP^32^ ATP showed that only IRAK1 IPed from IL-33 stimulated cells phosphorylated WT IRAK-M, but not the S110A mutant (Supplementary Fig 2f). These results support the idea that IRAK1 might phosphorylate IRAK-M in the Myddosome, as suggested by our NMR structure and docking model. This idea is consistent with our above observation that the IRAK-M DD is essential for IRAK-M pull-down by GST-PIN1.

To investigate whether PIN1 interaction with IRAK-M upon IL-33 stimulation induces release of IRAK-M from TLR complexes, we examined the effects of PIN1 on IRAK-M nuclear translocation in DC2.4 cells. Before IL-33 stimulation, IRAK-M was predominantly in the cytoplasm, whereas PIN1 was located in the nucleus (Supplementary Fig 3a**)**. Upon IL-33 induction, IRAK-M was also evident in the nucleus where it associated with PIN1 (Supplementary Fig 3a**)**. These results were further confirmed by DC2.4 cellular fractionation (Supplementary Fig 3b**)** and by immunostaining of CD205^+^ macrophages obtained from BALF of WT mice lungs treated with intranasal IL-33 (Supplementary Fig 3c**)**. Interestingly, the S110 phospho-mimicking mutant, S110E IRAK-M, showed higher nuclear localization (Supplementary Fig 3d), which was evident in *PIN1* KO MEF’s only after PIN1 reintroduction in these cells (Supplementary Fig 3e). Thus, IRAK-M nuclear localization seems to be dependent on S110 phosphorylation and on PIN1.

**Fig. 4:**
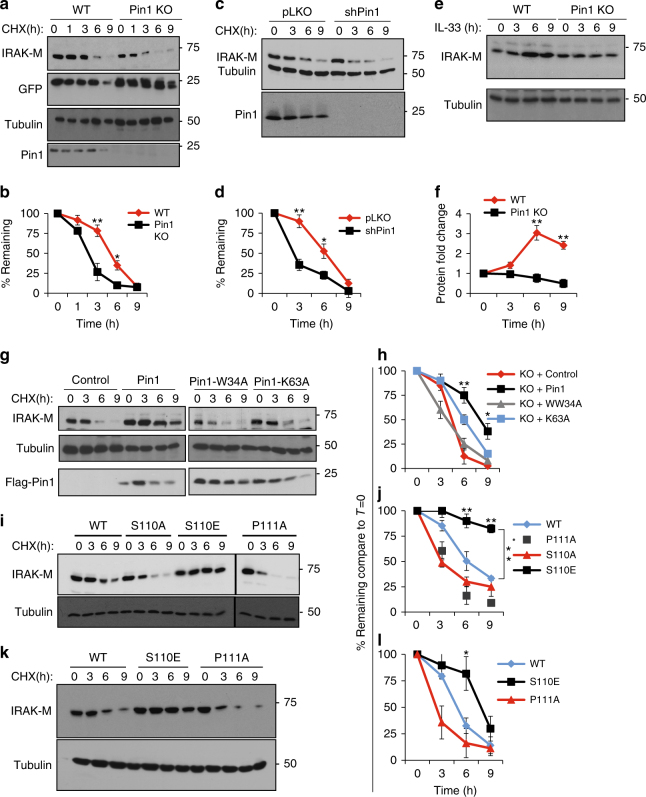
PIN1 increases IRAK-M protein stabilization. **a** After IRAK-M and GFP were coexpressed in WT and *PIN1* KO MEFs for 24 h, cells were split equally into 5 dishes. 24 h later cells were treated with cyclohexamide and harvested to monitor IRAK-M, GFP and PIN1 levels at the time points indicated. **b** Quantification of 3 independent experiments as in **a**. **c** WT MEFs stably expressing the TET on inducible short hairpin for *PIN1* (shPin1) or pLKO as a control, were expressed with IRAK-M, followed by induction with Doxycycline for 18 h prior to the cyclohexamide chase. **d** Quantification of 3 independent experiments as in **c**. **e** BMDCs from WT or *PIN1* KO mice were treated with IL-33 and the levels of IRAK-M were monitored at different time points after induction. **f** Quantification of 3 independent experiments as in **e**. **g** *PIN1* KO MEFs were expressed with IRAK-M alone or in combination with PIN1 or its mutants W34A or K63A for 24 h, followed by the cyclohexamide chase to assay IRAK-M stability. **h** Quantification of 3 independent experiments as in **g**. **i** IRAK-M or its different mutants; S110A, S110E and P111A were expressed in WT MEFs, followed by the cyclohexamide chase to assay IRAK-M stability. **j** Quantification of 3 independent experiments as in **i**. **k** IRAK-M or its different mutants were stably expressed in DC2.4 cells, followed by the cyclohexamide chase to monitor IRAK-M stability. **l** Quantification of 3 independent experiments as in **k**. The data were analyzed by a Student’s two-tailed *t* test and the values are reported as mean ± standard errors of the means (SEM). *- statistical significance (*P* < 0.05), **- significance (*P* < 0.01)

Since PIN1 can both promote nuclear import and increase stability of its substrate proteins^21,44,45^, we investigated the effects of PIN1 on IRAK-M protein stability. IRAK-M was co-overexpressed in WT and *PIN1* KO MEF’s with GFP as a control, followed by monitoring IRAK-M protein stability using cycloheximide chase. The absence of PIN1 reduced the IRAK-M half-life from ~6 h to <3 h (Fig. 4a-b), without affecting control GFP (Fig. 4a). These results were confirmed by the findings that inducible PIN1 knock down (KD) reduced IRAK-M half-life by more than 50% (Fig. 4c-d).

Given that IL-33 induces PIN1 to act on IRAK-M, we investigated the effects of IL-33 stimulation on the ability of PIN1 to regulate IRAK-M levels. Bone marrow dendritic cells (BMDC) derived from WT and *PIN1* KO mice were treated with IL-33 for different times, followed by assaying IRAK-M protein levels. IL-33 increased IRAK-M protein levels in WT, but not *PIN1* KO cells in a time-dependent manner (Fig. 4e-f). For further validation, we co-overexpressed IRAK-M in PIN1 KD MEFs with PIN1 or PIN1 binding or isomerization defective mutants W34A or K63A, respectively (Fig. 4g-h). PIN1 overexpression indeed increased IRAK-M half-life by more than 50%, whereas neither binding nor isomerizing mutants had any effect.

To investigate the dependence of IRAK-M protein stability on the pS110P motif, the S110A, P111A and S110E IRAK-M mutants were examined. While the S110A and P111A mutations decreased IRAK-M protein stability by ~50%, the S110E mutation rendered IRAK-M completely resistant to degradation in WT MEFs (Fig. 4i-j), which was also reproduced in DC2.4 cells (Fig. 4k-l). Thus, upon PIN1 binding and isomerization of the pS110P motif, IRAK-M may dissociate from the Myddosome, leading to its nuclear localization and protein stability.

### PIN1 regulates IRAK-M and IL-33 allergic response in mice

Given the dramatic impact of PIN1 on IRAK-M nuclear translocation and protein stability upon IL-33 stimulation in vitro, we asked whether endogenous PIN1 is critical for IRAK-M expression and inflammation upon IL-33 challenge in vivo using WT and *PIN1* KO mice. These mice were treated intranasally with IL-33, and IRAK-M immunostaining was performed to detect IRAK-M protein levels in bronchial epithelial cells and immune infiltrating cells, the most abundant IRAK-M expressing cells in the lung^33^. Intranasal IL-33 administration dramatically increased IRAK-M expression in these cells, and caused severe lung inflammation and bronchial remodeling (Supplementary Fig 4a-b). More importantly, both phenotypes were largely attenuated in the *PIN1* KO mice.

### IRAK-M is crucial for expression of T_H_2 regulatory genes

**Fig. 5:**
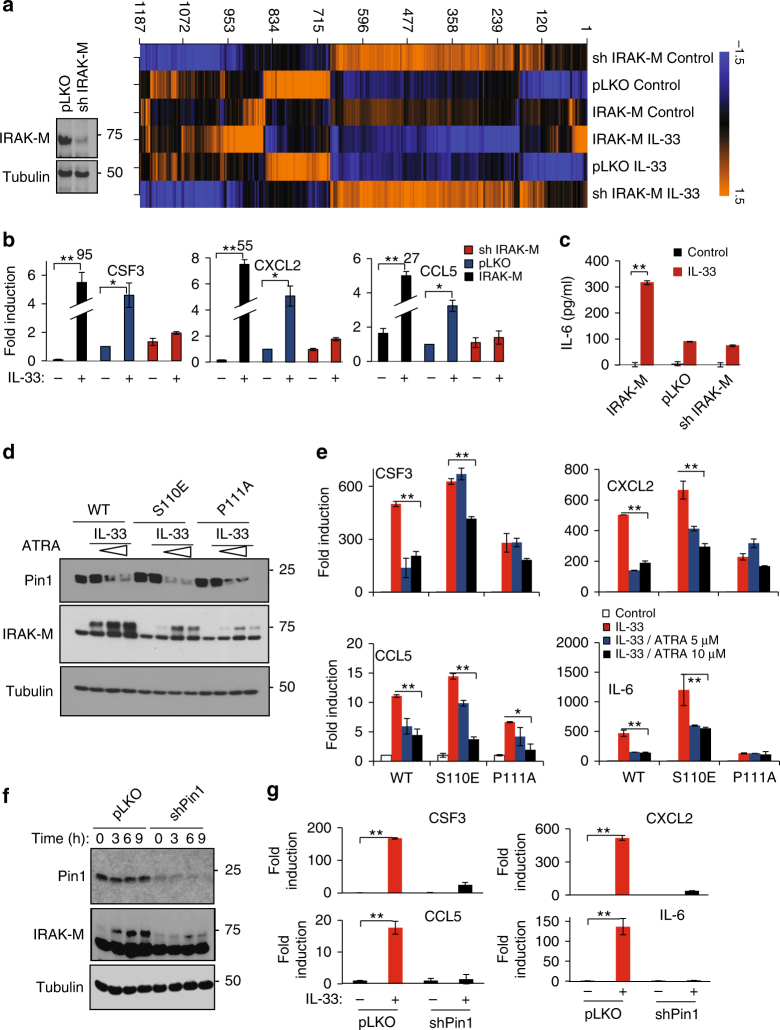
IRAK-M is necessary for *Il6*, *Csf3*, *Cxcl2* and *Ccl5* expression in dendritic cells upon IL-33 induction. **a** Western blot shows IRAK-M levels in shIRAK-M expressing DC2.4 cells. Right panel shows heat map showing expression levels of different genes according to the affymetrix gene expression profiling analysis. **b** qRT-PCR analysis for expression of the indicated genes normalized to actin. The numbers over bars (95, 55 and 27) indicate the folds of induction of target genes in the presence vs absence of IL-33 in the sh IRAK-M group. **c** Quantification of IL-6 release measured by ELISA. **d** IRAK-M, IRAK-M S110E or IRAK-M P111A stably expressing DC2.4 cells were either treated or not with IL-33. Also as indicated, some cells were pretreated for 3 days with 5 μM or 10 μM of ATRA before IL-33 induction. **e** Relative gene expression of *Il6*, *Csf3*, *Cxcl2* and *Ccl5* in the different samples as in **d** normalized to actin. **f** IRAK-M was stably expressed in pLKO or TET on shPin1 expressing cells, followed by induction with IL-33, before western blot to examine protein expression of IRAK-M, PIN1 and tubulin as well as **g** qRT-PCR to determine the relative gene expression for *Il6*, *Csf3*, *Cxcl2* and *Ccl5* in the different samples. The data were analyzed by a Student’s two-tailed *t* test and the values are reported as mean ± standard errors of the means (SEM). *- statistical significance (*P* < 0.05), **- significance (*P* < 0.01)

In addition to the known inhibitory function of IRAK-M in TLR/IL-1R signaling^31^, it has also been reported that IRAK-M is crucial for NF-κB activation in *Irak1* and *Irak2* double KO mice^46^. Our above results uncover an unexpected positive role of IRAK-M and its regulation by PIN1 in IL-33-induced inflammation. A critical question is what are the IRAK-M downstream mediators. Our results showing IRAK-M localized to the nucleus upon IL-33 stimulation prompted us to profile the effects of IL-33 treatment and IRAK-M levels on gene expression in DC2.4 cells. Control pLKO-expressing DC2.4 cells, IRAK-M KD cells (Fig. 5a) and IRAK-M stably overexpressing cells were treated with IL-33, followed by genome-wide gene expression profiling using GeneChip *Mouse* Genome *430* 2. The status of IRAK-M expression conferred distinct transcriptomes to DC2.4 cells (Fig. 5a). Among the genes whose expression showed strong dependence on IRAK-M after IL-33 challenge, are *Csf3*, *Cxcl2*, *Il6* and *Ccl5* (Fig. 5b-c). These genes were previously shown to be involved in the development of T_H_2 type response and upregulated in pulmonary disorders^47–50^. Ingenuity Pathway Analysis of the differentially expressed genes revealed major perturbations in key DC pathways, including the IL-6 and T helper cell differentiation pathway (Supplementary Fig 5a).

To validate these differentially expressed genes, we performed RT-PCR analysis. Notably, upon IL-33 treatment, expression of these four genes was upregulated in pLKO control DC2.4 cells, which was attenuated or blocked by IRAK-M KD (Fig. 5b-c). In contrast, IRAK-M stable overexpression drastically elevated their expression (Fig. 5b-c). These findings were further confirmed in IL-33-challenged BMDCs derived from WT and *IRAK-M* KO mice (Supplementary Fig 5b).

Given our findings that PIN1 regulates IRAK-M, we asked whether these IRAK-M dependent expression effects are dependent on PIN1. To address this question, we evaluated the effects of PIN1 inhibition and IRAK-M mutations at the pS110-P site on the expression of these four target genes. If their IRAK-M-dependent expression is mediated by PIN1 isomerization of the pS110-P site, then their expression would be minimally altered by PIN1 inhibition when employing IRAK-M mutants lacking the pS110-P motif. To this end, we treated DC2.4 cells stably expressing IRAK-M or two mutants (S110E as a constitutive PIN1 substrate or P111A as a non-substrate of PIN1) with increasing doses of the PIN1 inhibitor ATRA^51^ before IL-33 treatment. Without ATRA treatment, the S110E mutant elevated expression of these target genes to levels comparable to those observed in IRAK-M expressing cells, while the P111A mutant had a much weaker effect (Fig. 5d-e). Importantly, ATRA treatment not only reduced PIN1 expression (Fig. 5d-e), but also inhibited target gene expression in IRAK-M stably expressing cells, even at the lower ATRA dose (5 µM) (Fig. 5e). However, in both IRAK-M mutants, the effect of ATRA treatment on the expression of these genes was highly attenuated. With the S110E mutant, some reduction in gene expression still persisted at the higher ATRA dose (10 µM), which was completely abolished in P111A mutant (Fig. 5e). These results are consistent with our CO-IP experiments showing that PIN1 can still interact with the S110E mutant although at a lower extent, but completely fails to bind to the P111A mutant (Fig. 2i), and with our NMR results showing that PIN1 catalyzes isomerization of the IRAK-M-S110E peptide (Fig. 3c).

To further confirm these findings, we measured the expression of these target genes after IL-33 stimulation in IRAK-M-expressing DC2.4 cells where PIN1 is inhibited by genetic knockdown. *PIN1* KD partially prevented IRAK-M phosphorylation and completely abolished the effects of IRAK-M on expression of these four genes upon IL-33 treatment (Fig. 5f-g). For further confirmation, we stably expressed IRAK-M S110E mutant in *PIN1* KD DC2.4 cells and found that overexpression of S110E mutant could only partially restore the expression of these target genes, in the absence of PIN1, upon IL-33 stimulation (Supplementary Fig 5c). Thus, IRAK-M is crucial for the expression of these pro-inflammatory genes, and its phosphorylation on the S110-P motif and subsequent isomerization by PIN1 upon IL-33 challenge are necessary steps for the expression of these key target genes.

### PIN1-IRAK-M axis regulates IL-33 allergic response in mice

**Fig. 6:**
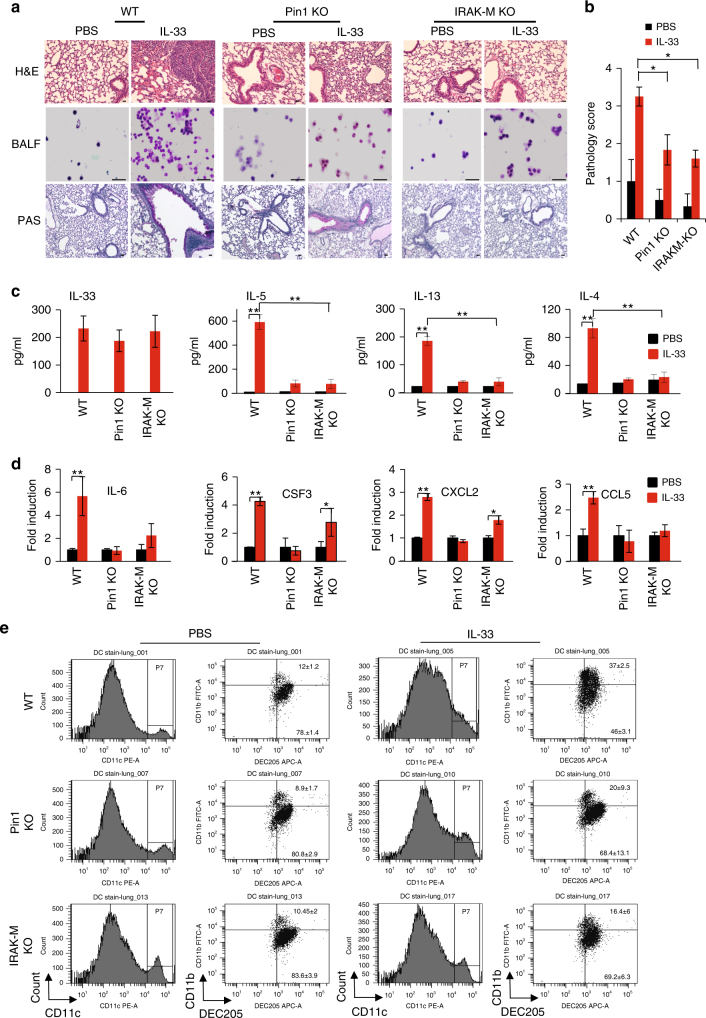
*IRAK-M* KO and *PIN1* KO dramatically reduce lung inflammation upon IL-33 treatment in vivo. **a** Representative H&E, PAS staining of lung sections from various treated mice, as well as BALF cytospin from the treated mice stained with Giemsa stain (*n* = 5). Scale bar = 50 µm. **b** Histopathology score- All H&E and PAS histological samples were examined and scored in a blind manner by the core facility pathologist (*n* = 5). **c** ELISA measurements of IL-33, -5, -13 and IL-4 in the BALF of the mice treated with PBS or IL-33. **d** RNA was obtained from the whole lung tissue and analyzed for the relative expression of *Il6*, *Csf3*, *Cxcl2* and *Ccl5* by qRT-PCR. **e** CD11c^+^ CD11b^+^ CD205^+^ cells were monitored after PBS or IL-33 challenge in the indicated mice (*n* = 3). In WT mice there was a 3-fold induction (P < 0.05) in CD11c^+^ CD11b^+^ CD205^+^ cells upon IL-33 treatment. *PIN1* KO mice showed a more moderate increase and *IRAK-M* KO mice showed non significant (NS) increase in the CD11c^+^ CD11b^+^ CD205^+^ cells upon IL-33 treatment. The data were analyzed by a Student’s two-tailed *t* test and the values are reported as mean ± standard errors of the means (SEM). *- statistical significance (*P* < 0.05), **- significance (*P* < 0.01)

To examine the importance of endogenous IRAK-M or PIN1 for IRAK-M target gene expression and inflammation upon IL-33 challenge, we treated WT, *PIN1* KO and *IRAK-M* KO mice with intranasal IL-33, followed by examining lung pathology, BALF cellular content, T_H_2 cytokines, and expression of IRAK-M downstream targets in lung tissues. WT mice exhibited high inflammation, as evident by the high presence of inflammatory cells surrounding the bronchoalveolar space and strong PAS staining of the bronco-epithelial cells (Fig. 6a). Cytospin of BALF cells from WT mice showed elevation in the number of total cells, mostly in granulocytes such as neutrophils and eosinophils (Fig. 6a). These effects were highly attenuated in both *PIN1* KO and *IRAK-M* KO mice, reflected by the pathology score of these sections (Fig. 6a-b). Different BALF cytokine measurements showed that while high levels of IL-33 were detected in all IL-33 treated groups, WT mice exhibited high levels of T_H_2 cytokines (IL-4, -5 and -13), but these cytokines were dramatically attenuated in *PIN1* KO or *IRAK-M* KO mice (Fig. 6c). For further confirmation, CD3^+^ CD4^+^ T cells were FACS sorted for RNA isolation (Supplementary Fig 6a-b), and *Il13* and -*5* expressions from these cells were monitored (Supplementary Fig 6c). As expected, IL-33 markedly induced expression of *Il13* and -*5*, which was attenuated in *PIN1* KO or *IRAK-M* KO mice. Expression of all four IRAK-M downstream targets was upregulated in WT mice upon IL-33 stimulation, with *Il6* and *Csf3* showing the most predominant effect (Fig. 6d), and their expression was attenuated in *PIN1* KO or *IRAK-M* KO mice (Fig. 6d).

It has been reported that IL-33-activated DCs are necessary for airway inflammation^15^. To examine whether *PIN1* KO or *IRAK-M* KO has any effect on DC activation in the lung upon IL-33 stimulation, WT, *PIN1* KO and *IRAK-M* KO mice were treated as before. Lungs were dissected and the expression of CD11b^+^ surface markers of activated DC cells were monitored (CD11c^+^ CD205^+^ CD11b^+^). We chose to focus on CD11b^+^ DCs since they are responsible for T_H_2 priming^52–54^. IL-33 challenge induced the CD11b^+^ expressing DCs in WT treated mice after IL-33 treatment, which was attenuated in *PIN1* KO or *IRAK-M* KO mice (Fig. 6e and Supplementary Fig 6d).

Previously it was reported that treatment of DCs with IL-33 upregulates the expression of costimulatory proteins CD40 and CD80, which correlates with DC activation and induction of IL-5 and IL-13 production from co-cultured naive CD4+ T cells^15,17^. To investigate the dependence of these effects on IRAK-M, lung CD11c^+^ cells from WT and *IRAK-M* KO mice, either treated with IL-33 or PBS as before, were evaluated for the expression of their costimulatory proteins CD40, CD80 and CD86. Upon IL-33 stimulation, the expression of both CD40 and CD80 were elevated in CD11c^+^ cells obtained from IL-33 treated WT mice, whereas this overexpression was attenuated in lung CD11c^+^ cells obtained from IL-33 treated *IRAK-M* KO mice (Supplementary Fig 6e).

We next investigated whether IRAK-M and/or PIN1 are required for IL-33-induced T_H_2 polarization. We co-cultured WT, *IRAK-M* or *PIN1* KO mouse BMDCs with WT naive CD4^+^ cells, followed by IL-33 stimulation. The naive CD4^+^ cells secreted high levels of IL-5 and -13 when co-cultured with BMDCs in the presence of IL-33 (Supplementary Fig 7a-b). However, this effect was significantly attenuated when *IRAK-M* KO BMDC (Supplementary Fig 7a) or *PIN11* KO BMDC (Supplementary Fig 7b) were used, or when PIN1 was inhibited by ATRA. Thus, loss of either IRAK-M or PIN1 disrupts IL-33 induced secretion of IL-5 and IL-13, further supporting our hypothesis that the PIN1-IRAK-M axis is necessary for DC activation, T_H_2 polarization and inflammation upon IL-33 challenge.

### Overexpression of IRAK-M and its targets in asthma patients

**Fig. 7:**
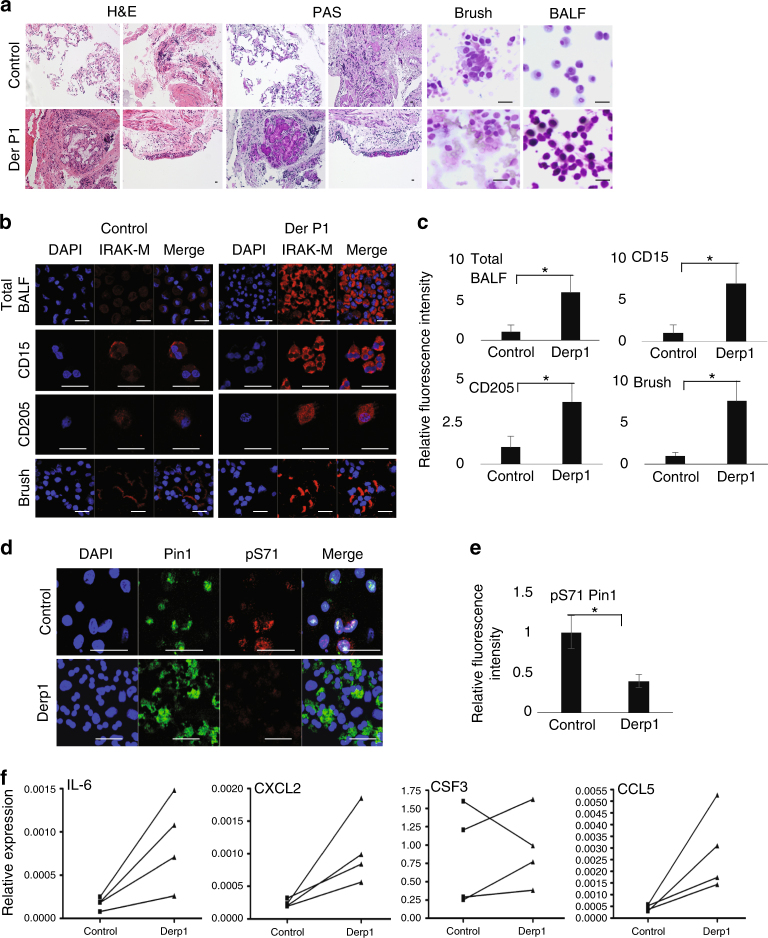
IRAK-M expression is elevated in asthmatic human samples after Derp1 segmental allergic challenge. **a** Representative H&E staining and PAS staining of lung biopsies before and after Derp1 segmental challenge, as well as BALF and brush cytospins before and after treatment stained with Giemsa (*n* = 4). **b** Cytospin slides of brushing, total BALF cells, BALF CD15 + and CD205 + cells samples were immune stained for IRAK-M. **c** Quantification of IRAK-M expression in total BALF cells, BALF CD15 + and CD205 + cells samples as in **b** using Velocity program. **d** Total BALF cells were immune stained for PIN1 and pS71PIN1 and quantified using Velocity program **e**. **f** RNA was extracted from BALF cellular contents and analyzed for the relative expression of *Il6*, *Csf3*, *Cxcl2* and *Ccl5* by qRT-PCR. The data were analyzed by a Student’s two-tailed *t* test and the values are reported as mean ± standard errors of the means (SEM). *- statistical significance (*P* < 0.05), **- significance (*P* < 0.01). Scale bar = 50 µm

To investigate whether our findings correlate with samples from human participants, we assessed levels of IRAK-M protein and its upstream and downstream targets in asthmatic patients using a segmental allergen Derp1 challenge, a well-known induction of allergic asthma in humans^55,56^. Derp1 treatment caused a massive immunological reaction, which was evident by the infiltration of granulocytes into the lung tissue and tissue remodeling of the bronchial epithelial cells as was evident by PAS tissue staining (Fig. 7a). To evaluate IRAK-M protein levels and localizations, we immune stained our different samples for IRAK-M. In fixed biopsy section samples obtained from patients before and after Derp1 challenge (Supplementary Fig 8**)**, IRAK-M expression was elevated in the bronchial epithelial cells and predominantly located to the apical side of the cells (Fig. 7b-c). In the tissue sections, we were able to locate more cells expressing IRAK-M than in the control biopsies, which correlated with residence/infiltrating immune cells.

IRAK-M immunostaining of brush and total BALF cytospin samples before and after challenge showed that IRAK-M protein expression was greatly elevated in the lung brush samples obtained after Derp1 challenge (Fig. 7b-c). In total BALF samples, IRAK-M expression was evident before challenge and was highly elevated in different cell types after Derp1 challenge **(**Fig. 7b-c**)**. To better examine IRAK-M expression in different cell types, BALF samples were cell sorted using CD15^+^ and CD205^+^ markers for eosinophils and dendritic/monocytes, respectively (Fig. 7b-c). In both cases IRAK-M expression was highly elevated after Derp1 treatment. We also monitored PIN1 and its inhibitory S71 phosphorylation by immunostaining of total BALF cytospins. PIN1 protein expression did not seem to change upon Derp1 treatment, but we did observe a decrease in pS71 phosphorylation (Fig. 7d-e), consistent with the previous findings that PIN1 catalytic activity is elevated in the airway of human asthma patients^25^.

Finally, we evaluated the expression of IRAK-M target genes in the BALF samples. We detected an overall upregulation in the expression of *Il6*, *Cxcl2* and *Ccl5* in all four samples, although the results for *Cs*f3 expression were more variable, especially in one patient (Fig. 7f). Collectively, these results from asthma-induced human samples correlate with our findings obtained using mouse models, showing that IL-33-induced upregulation in the expression of IRAK-M is associated with upregulation of proinflammatory IRAK-M dependent genes.

## Discussion

IRAK-M is traditionally considered as a negative regulator of TLR/IL-1R signaling^31^. It is thought to do so by trapping IRAK1 in the Myddosome and preventing its downstream signaling. This idea has been corroborated by in-vivo data showing *IRAK-M* KO macrophages produce higher cytokine levels in response to some TLR agonists and that *IRAK-M* KO mice challenged with influenza virus show exaggerated immunological responses which are accountable for higher mortality rate and enhanced early influx of cytotoxic T cells and lung epithelial tissue damage^57^. In contrast, other reports indicate that IRAK-M may act as an inducing mediator in the TLR/IL-1R signaling pathway. Notably, overexpression of IRAK-M in HEK293 cells activates NF-κB, an effect that is dependent on the IRAK-M N-terminal DD domain^58^. Overexpression of IRAK-M in *IRAK1* KO cells is sufficient to restore IL-1-induced NF-κB activation^58^, indicating that IRAK-M and IRAK1 may exert similar functions under certain stimulations. Moreover, IRAK-M may mediate TLR/IL-1R activation of NF-κB in the absence of IRAK1 and IRAK2, in a MEKK3 dependent manner^46^. Finally, IRAK-M may activate the NF-κB pathway and cell responsiveness upon IL-1 stimulation while having the opposite effect upon TLR 4 stimulation^59^. These contradictory functions of IRAK-M could indicate that IRAK-M may function as an on/off switch for the immune response, in a specific stimulation-dependent manner, based on the specific phosphorylation status of IRAK-M.

**Fig. 8:**
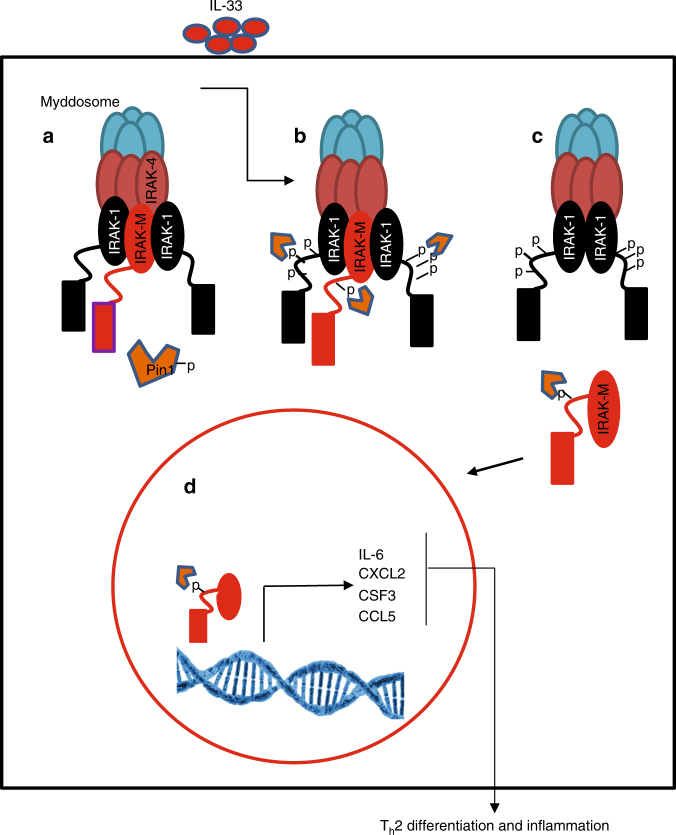
Proposed model for IRAK-M activation mechanism by IRAK1 and PIN1, upon IL-33 stimulation. **a** IRAK-M is located in the Myddosome in association with IRAK1. PIN1 is phosphorylated at Ser71 and is inactive. **b** Upon IL-33 stimulation, IRAK1 is hyper phosphorylated and active and phosphorylates IRAK-M at S110P site. PIN1 is dephosphorylated and active. **c** PIN1 binds to IRAK-M phosphorylation site and induces conformational change that promotes its dissociation from the Myddosome. **d** Released IRAK-M translocates into the nucleus and induces the expression of its target genes for downstream T_H_2 cell differentiation and inflammation

Here we show that IRAK-M has a distinctive function upon IL-33 stimulation where it serves as a significant mediator for IL-33 signaling in DCs. IL-33 stimulation of DCs increases IRAK-M levels, activates PIN1, and induces IRAK-M phosphorylation at its S110-P site by IRAK1, thereby making IRAK-M a putative substrate for PIN1 isomerase regulation. Subsequently, PIN1 binding and isomerization of the pS110P motif increases IRAK-M nuclear translocation and protein stability that correlates with increased expression of a set of pro-inflammatory cytokines that contribute to inflammation (Fig. 8). Moreover, genetic ablation of PIN1 or IRAK-M, or inhibition of PIN1 by ATRA, attenuates T_H_2 polarization, DC activation and overall lung inflammation upon IL-33 challenge in mouse models. These results were correlated with molecular features of asthma in humans by analyzing samples obtained from Derp1 challenged asthmatic patients, where we detected increases in PIN1 activation and IRAK-M levels, and increased expression of the IRAK-M downstream pro-inflammatory cytokines.

These biochemical and functional results were consistently observed in cell lines, in mouse models, and in primary cells from mouse models, and were correlated with analyses of human patient samples. These findings demonstrate a central function of IRAK-M and PIN1 in IL-33 induced inflammation. Our high-resolution NMR structure of the IRAK-M-DD and subsequent simulations of docking of this domain into the Myddosome suggests a model to explain how IRAK-M could participate in IL-33 induced pro-inflammatory signaling, and how PIN1 could regulate this process. According to this model, IRAK-M phosphorylation by IRAK1 and subsequent binding and isomerization by PIN1 displaces the C-terminal region of the IRAK-M DD, thereby disrupting the N-to-C interaction and destabilizing the bound IRAK-M DD subunits in the Myddosome, inducing the dissociation of IRAK-M from the Myddosome complex. This displacement may allow IRAK-M to be shuttled to the nucleus (Fig. 8). The precise mechanisms by which IRAK-M stability and nuclear translocation are achieved will require further study.

IRAK-M can shuttle to and out of the nucleus^60^. Although we show that IL-33 challenge causes PIN1-dependent nuclear translocation of IRAK-M, the precise role of IRAK-M in the nucleus has not yet been reported. Given the correlation observed here between IRAK-M expression and the upregulation of several genes, it is tempting to speculate that IRAK-M might induce gene expression by an as yet unknown mechanism. Our genome wide expression profiling has identified four genes (*Il6*, *Cxcl2*, *Csf3* and *Ccl5*) whose expression is dependent on the IL-33-IRAK-M-PIN1 axis, and IRAK-M phosphorylation at Ser 110. These four IRAK-M target genes have been shown to be crucial for asthma and other pulmonary disorders^47–50^ and their roles in granulocytes function have been documented. Specifically, CXCL2 is involved in neutrophil and basophile chemotaxis and degranulation as well as in normal and asthmatic airway smooth muscle cell migration^49^. IL-6 promotes T_H_2 differentiation of CD4^+^T cells while suppresses Th1 differentiation^61^. CSF3 controls the production, differentiation, and function of granulocytes such as neutrophils and eosinophils^62^. It has also been reported that CSF3 expression in the lung is correlated with severity of pulmonary neutrophilia in acute respiratory distress syndrome^63^. CCL5 serves as chemoattractant for monocytes, memory T helper cells and eosinophils, as well as the activation of eosinophils^64^ and is highly expressed in respiratory secretions of asthmatic patients^47^. Indeed, we have confirmed the upregulation of these genes in samples from asthmatic human patients induced with allergen. Thus, the potent ability of the herein established IL-33-IRAK-M-PIN1 axis to induce this set of pro-inflammatory cytokines may contribute to their critical roles in asthma.

## Methods

### Cell lines and reagents

The DC2.4 cell line (catalog number SCC142, Merck), derived from C57BL/6 bone marrow, was kindly provided by Dr. Kenneth Rock (University of Massachusetts Medical Center, Worcester, MA). Cells were grown in complete media comprised of DMEM, supplemented with 10% FBS, 10 mM HEPES, 2 mM L-glutamine and 50 µg/ml gentamicin. Wild type (*Pin1*^+/+^) and *PIN1* KO (*Pin1*^-/-^) mouse embryonic fibroblasts (MEFs) were available at our laboratory. WT and *PIN1* KO MEFs were maintained in complete media comprised of DMEM, supplemented with 10% FBS, 2 mM L-glutamine. Bone marrow dendritic cells (BMDC) were prepared as fallow: BMDC were extracted from tibias and femurs and were cultured in the presence of 10 ng/ml GM-CSF (PeproTech) for 10 days. All cells were maintained at 37 °C in a humidified incubator with 5% CO_2_. Cells were maintained via weekly passage and utilized for experimentation at 60–80% confluency. Rabbit Polyclonal anti IRAK-M was purchased from Sigma (catalog number SAB3500193) and was used according to manufacture instructions (for WB 1:5000; for IP 1:400; for IF: 1:200). Rabbit monoclonal Anti PIN1 was purchased from EPITOMICS (catalog number ab76309**)** and was used according to manufacture instructions (for WB 1:2000; for IP 1:500; for IF: 1:200). Recombinant mouse IL-33 was purchased from BioLegend (catalog number 580506). IL6 (catalog number 50-112-8863),-5(catalog number 50-112-5185),-4(catalog number 50-112-8931) and -13(catalog number 50-112-8703) ELISA kits Ready-Set-Go was from eBioscience. shRNA for IRAK-M was purchased from DANA Farber siRNA core facility.

### PIN1 activity assay

DC2.4 cell line were either treated with 100ng/ml IL-33 for indicate time (0, 5 and 15 min). The cells were harvested and homogenized in a reaction buffer containing 100 mM NaCl, 50 mM HEPES, pH 7, 2 mM DTT, and 0.04 mg/ml BSA. The lysates were cleared by centrifugation at 12,000 *g* for 10 minutes (4 °C). PPIase activity was measured using equal amounts of parathyroid cytoplasmic lysates and α-chymotrypsin using a synthetic tetrapeptide substrate Suc-Ala-Glu-Pro-Phe-pNa (Peptides International). Absorption at 390 nM was measured using an Ultrospec 2000 spectrophotometer. The results are expressed as the mean of 3 measurements from a single experiment and are representative of 3 independent experiments.

### GST pulldown assay and immunopercipitation

Glutathione-Sepharose 4B (Amersham) coupled with glutathione *S*-transferase (GST) or GST-PIN1 and washed with 150 mM NaCl, 20 mM Tris-HCl (pH 8), 1 mM MgCl_2_, and 0.1% NP-40 was mixed with 1 mg of total protein extract for 3 h at 4 °C. The beads were then washed extensively, and the protein was eluted with 20 mM reduced glutathione. The proteins were resolved by SDS-PAGE and visualized by Western analysis or autoradiography. For tandem mass spectrometry analysis the eluted protein were further immunopercipited for IRAK-M. In this case, the eluted proteins were incubated with pre incubated immobilized Protein A agarose beads (RepliGen) premixed with IRAK-M antibody, for three hours. The beads were extensively washed with lysis buffer containing 150 mM NaCl, 1% NP-40, 0.5% deoxycholate, 0.1% SDS, 50 mM Tris-HCl (pH 7.7), 1 mM phenylmethylsulfonyl fluoride (PMSF), 1 mM dithiothreitol, and protease inhibitors before elution in SDS sample buffer.

### Coimmunoprecipitation

Cells from one 10-cm dish were homogenized in lysis buffer containing 150 mM NaCl, 1% NP-40, 0.5% deoxycholate, 0.1% SDS, 50 mM Tris-HCl (pH 7.7), 1 mM phenylmethylsulfonyl fluoride (PMSF), 1 mM dithiothreitol, and protease inhibitors. Clarified supernatants were incubated with pre incubated immobilized beads rProtein A agarose beads (RepliGen) premixed with PIN1, GFP or IRAK-M antibody or, as a control, IgG overnight at 4 °C. The beads were washed extensively with lysis buffer before elution in SDS sample buffer.

### Animal handling and asthma induction

*PIN1* knockout mice have been backcrossed to C57L/B6 for 15 generations, with *PIN1*^-/-^ and *PIN1*^+/+^ littermates were used in this study. *IRAK-M* KO mice were a kind gift from Prof. Koichi S Kobayashi^31^. All mice were maintained in specific pathogen free (SPF) unit at Beth Israel Deaconess Medical Center, Boston, MA, USA.

Male mice in each group, 3-5 weeks old were anesthetized using Isoflurane. The mice were treated intranasal with 200ng/mice/day for four continues days. By the fifth day the mice were killed by CO2 suffocation, and bronchial alveolar lavage fluid (BALF) was extracted using PBS. The lungs were extracted and fixed using 10% para formaldehyde. Immunofluresence of slide sections was performed. Slides were analyzed using anti-PIN1 antibodies and anti-IRAK-M antibodies or IgG as control and were counterstained using DAPI. In some cases, cells obtained from BALF were cytospined and the cells were fixed and stained in the same manner. For staining of the cells, Diff-Quick (Diff-Quik) Staining Protocol were used. All animal work was carried out in compliance with the ethical regulations approved by the Animal Care Committee, Beth Israel Deaconess Medical Center, Boston, MA, USA. The permission was granted to perform animal experiments by the Institutional Animal Care and Use Committee at Beth Israel Deaconess Medical Center, Boston, MA, USA.

### ELISA measurements

ELISA measurements carried out using eBioscience ELISA Ready-Set-Go system according to manufacture protocol.

### Flow analysis of dendritic cells and T cells and cytospins

Lungs were digested using Kollagenase D and dispase and the dendritic cells populations (CD11c^+^ CD205^+^ CD11b^+^) were determined by staining the cells using the following antibodies: PE anti mouse CD11c (catalog number 117307), APC anti mouse CD205 (catalog number 138205), FITC anti mouse CD11b (catalog number 101205). For co stimulatory protein expression, FITC anti mouse CD40 (catalog number 124607), FITC anti mouse CD80 (catalog number 104705), FITC anti mouse CD86 (catalog number 105005) were used. For T cell (CD3^+^ CD4^+^) population isolation, the cells were stained with APC anti mouse CD3^+^ (catalog number 100235) FITC anti mouse CD4^+^ (catalog number 100405) before the cells were cell sorted. Human BALF samples were stained with FITC anti human CD15 (catalog number 301903) or FITC anti human CD205 (catalog number 342206). All antibodies were purchased from Biolegend and diluted according to manufacture instruction (1:100 dilution). Cells were analyzed using LSRII flow cytometer and FlowJo software. For cytospin the cells were spun onto glass slides, air dried and fixed using 4%PFA and stained with the indicated antibodies.

### Mass spectrometry analysis

For all mass spectrometry (MS) experiments, IRAK3 immunoprecipitates were separated using SDS-PAGE, the gel was stained with Coomassie blue, and the IRAK3 band was excised. Samples were subjected to reduction with DTT, alkylation with iodoacetamide, and in-gel digestion with trypsin overnight at pH 8.3, followed by reversed-phase microcapillary/tandem mass spectrometry (LC-MS/MS). LC-MS/MS was performed using an EASY-nLC II nanoflow HPLC (Thermo Scientific) with a self-packed 75 μm id x 15 cm C_18_ column connected to a high resolution Orbitrap Elite mass spectrometer (Thermo Scientific) in the data-dependent acquisition and positive ion mode at 300 nL/min. MS/MS spectra collected via CID were searched against the concatenated target and decoy (reversed) Swiss-Prot protein database using Mascot 2.4 (Matrix Science, Inc.) with differential modifications for Ser/Thr/Tyr phosphorylation (+79.97) and the sample processing artifacts Met oxidation (+15.99), deamidation of Asn and Gln (+0.984) and Cys alkylation (+57.02). Phosphorylated and non-phosphorylated peptide sequences were accepted as valid if they passed a 1.0 % false discovery rate (FDR) threshold. Passing MS/MS spectra were then manually inspected to be sure that all b- and y- fragment ions aligned with the assigned sequence and putative phosphorylation sites. Determination of the exact sites of phosphorylation was aided using Scaffold 4 and ScaffoldPTM software (Proteome Software, Inc.)

### Gene expression profiling

Gene expression was assessed using Affymetrix (Santa Clara, CA) GeneChip *Mouse* Genome *430* 2.0 arrays. 15 μg cRNA was fragmented and hybridized to arrays’ according to the manufacturer’s protocols. The quality of scanned array images were determined on the basis of background values, percent present calls, scaling factors, and 3’/5’ ratio of β-actin and GAPDH. Data were extracted from CEL files and normalized using RMAexpress (http://rmaexpress.bmbolstad.com/) and annotated using MeV software (http://www.tm4.org/mev.html). Differentially expressed genes between different conditions were determined using a fold change threshold of 2.

### Pathway and functional analysis

Pathway and Functional analyses of the differentially expressed genes were performed using the commercial systems biology oriented package, Ingenuity Pathways Analysis (www.ingenuity.com). IPA provides a framework by which the lists of genes identified by large microarray experiments can be annotated in terms of functional relationships to understand the underlying biological mechanisms. It calculates the p-value using Fisher’s Exact Test for each pathway and functions according to the fit of user’s data to IPA databases. The p-value measures how likely the observed association between a specific pathway/function and the dataset would be if it were only due to random chance, by also considering the total number of Functions/Pathways/Lists of eligible genes in the dataset and the Reference Set of genes (those which potentially could be significant in the dataset). In case of interactive networks, all the identified genes were mapped to genetic networks available in the Ingenuity database and were ranked by the score. The Score (-log *p*-value) is calculated using Fisher’s Exact Test and indicates the likelihood a gene will be found in a network due to random chance. For example, if a network achieves a score of 2, it has at least 99% confidence of not being generated by chance alone.

### Quantitative real-time PCR

Validation of differentially expressed genes was performed by RT-PCR. 200 ng of high quality RNA samples were reverse transcribed to first strand cDNA and 1 μl cDNA was used for each RT-PCR reaction. Samples were performed in triplicates. SYBR Green PCR Master Mix (Applied Biosystems, Foster City, CA) was used for two-step real-time RT-PCR analysis on an Applied Biosystems StepOnePlus Real Time PCR instrument. Primers’ sequences were designed using the rpimer3 tool (http://bioinfo.ut.ee/primer3-0.4.0/primer3/). Expression value of the targeted gene in a given sample was normalized to the corresponding expression of Actin. The 2^–ΔΔCt^ method was used to calculate relative expression of the targeted genes. The primer sequences are as fallow: m*Cxcl2* forward: CACTCTCAAGGGCGGTCAAA; m*Cxcl2* reverse; CAGGTCAGTTAGCCTTGCCT; m*Ccl5* forward; GCAGTCGTGTTTGTCACTCG; m*Ccl5* reverse: GCAAGCAATGACAGGGAAGC; m*Actin* forward: ACACCCGCCACCAGTTCG; m*Actin* reverse: CCACGATGGAGGGGAATACAG; m*Csf3* forward: CGTTCCCCTGGTCACTGTC; m*Csf3* reverse: TAGGTGGCACACAACTGCTC; m*Il6* forward: CACGGCCTTCCCTACTTCAC: m*Il6* reverse: GGTCTGTTGGGAGTGGTATCC; h*Il6* forward: TCAATATTAGAGTCTCAACCCCCA; h*Il6* reverse: TTCTCTTTCGTTCCCGGTGG; h*Cxcl2* forward: GGCATACTGCCTTGTTTAATGT; h*Cxcl2* reverse: TCTCTGCTCTAACACAGAGGGA; h*Ccl5* forward: CAGTCGTCTTTGTCACCCGA; h*Ccl5* reverse: TCTTCTCTGGGTTGGCACAC; h*Actin* forward: CCGTTCCGAAAGTTGCCTTTT; h*Actin* reverse: CCGCTGGGTTTTATAGGGCG; h*Csf3* forward: GAGGAAGATCCAGGGCGATG; h*Csf3* reverse: AGCTTGTAGGTGGCACACTC.

### NMR analysis for IRAK-M-PIN1 interactions and catalysis

All NMR spectra were recorded at 25 °C on a Varian Inova 600 MHz spectrometer equipped with a ^1^H/^13^C/^15^N triple resonance probe with Z-axis gradient. The ^15^N- labeled PIN1 WW domain (PIN1 residues 1-50) was expressed and purified. The PIN1 gene was inserted into a pET28 vector with Kanamycin resistance as a fusion protein with an N-terminal His_6_-tag separated by a TEV cleavage site. Full length PIN1 was expressed in LB culture. The cells were induced at OD_600_ of 0.6~0.8 by adding 1 mM of final concentration of IPTG at 16 °C for 20 h Cells were harvested by centrifugation at 3500 g, and the cell pellet was dissolved in PIN1 wash buffer (50 mM Phosphate, 300 mM NaCl, 20 mM Imidazol, and 0.1 mM TCEP, pH 8.0). Lysozyme was added to dissolved cells, and sonicated on ice for 1 min in 50% pulses and repeated 4 times. Cell debris was removed by centrifugation, and supernatant was incubated in a column with 2 mL Ni-NTA agarose (QIAGEN) pre-equilibrated with wash buffer. The column was washed with 4 bed volumes of PIN1 wash buffer, and His_6_-tag PIN1 was eluted with 4 bed volumes of elution buffer (300 mM of imidazole added to PIN1 wash buffer). Synthetic peptides IRAK-M-pS110 (comprised of IRAK-M residues 103-124 with Ser110 phosphorylated, TNYGAVL(**pS)**PSEKSYQEGGFPNI), and IRAK-M-S110E (IRAK-M residues 103-124 with the S110E substitution), were purchased from Tufts University, Core Facility, Boston, MA.

The IRAK-M[1–119] gene was encoded into the pMAL-c2X vector with N-terminal fusion partner MBP, separated by a Factor XA cleavage site. The R56D and Y61D mutations were achieved using this vector, a single primer (forward primer, with mutations underlined: 5’ AGC AGC TGG CTG GAT GTT GAT CAT ATT GAA AAG GAT GTA GAC CAA GGT AAA AGT G 3’) and the BIO RAD-iProof High-Fidelity DNA Polymerase to generate expression plasmid pMAL-c2X encoding the MBP- IRAK-M[1-119:R56D,Y61D] fusion protein, with a Factor XA cut site between MBP and IRAK-M[1-119:R56D,Y61D]. To produce uniformly ^15^N-labeled MBP-IRAK-M[1-119:R56D,Y61D] in *E*. *coli*, transformed cells were grown in M9 minimal media with ^15^NH_4_Cl as the sole nitrogen source, and were induced at OD_600_ by adding 0.4 mM final concentration of IPTG at 25 °C for 20~22 h. Cells were harvested by centrifugation at 3500 g, the cell pellet was dissolved in IRAK-M wash buffer (20 mM Tris, 200 mM NaCl, 1 mM EDTA, and 0.2 mM TCEP, pH 7.4), and sonicated on ice. Cell debris was removed by centrifugation, the supernatant was incubated with 5 mL of Amylose Resin (BioLabs) pre-equilibrated with IRAK-M wash buffer. The column was washed with 4 bed volumes of IRAK-M wash buffer, and the fusion protein was eluted with 2 bed volumes of elution buffer (10 mM of Maltose added to IRAK-M wash buffer). Eluted fusion protein was dialyzed into Factor XA cleavage buffer (20 mM Tris-HCl, 100 mM NaCl, 2 mM CaCl2, pH = 8.0). Factor XA was added (50ul of 1ug/ul to 10 ml of fusion protein) and the solution was gently rocked at room temperature overnight. The cut protein products were concentrated to 2~3 ml, dialyzed into FPLC buffer (10 mM Tris, 20 mM NaCl, and 0.2 mM TCEP, pH 8.0) and loaded onto a size exclusion FPLC column (BioRad). Eluted fractions containing IRAK-M[1-119:R56D,Y61D] were pooled, concentrated, and TCEP was added to a total concentration of 2 mM. The pH was adjusted to 6.65 using 0.1 M HCl. IRAK-M[1-119:R56D,Y61D] concentration was measured by UV absorption at 280 nm (extinction coefficient = 25105 cm^-1^ M^-1^). Purity was verified by SDS-PAGE.

Nuclear magnetic resonance (NMR) experiments were performed on a Varian Inova 600-MHz spectrometer at 25 °C. NMR spectra were processed and analyzed using NMRPipe and Sparky software; T. D. Goddard and D. G. Kneller, SPARKY 3, University of California, San Francisco). The composite chemical shift change in the 2D ^1^H ^-15^N HSQC was monitored during NMR titration experiments and was fit to the standard bimolecular binding equation. Fitting of the data to the standard bimolecular binding equation was achieved using Excel and the Solver Add-in (Microsoft).

To quantify the binding affinity between the PIN1 WW domain and peptides, the ^15^N labeled PIN1 WW domain was titrated with the each of the synthetic peptides, IRAK-M-pS110 and IRAK-M-S110E. A reverse titration method was used, where the ^15^N labeled protein was mixed with a high concentration synthetic peptide for the first sample. Subsequent samples were a serial dilution of this sample with one part derived from the previous sample and one part from a stock solution of the ^15^N labeled PIN1 WW domain at the same concentration as the ^15^N labeled PIN1 WW domain in the first sample. This resulted in a titration where the concentration of the^15^N PIN1 WW domain was constant, and the concentration of synthetic peptide decreased by a factor of 0.5 in each successive sample. For each of the IRAK-M-pS110 and IRAK-M-S110E peptides, a ^1^H ^-15^N HSQC of ^15^N-PIN1-WW at each titration point was acquired on a Varian Inova 600-MHz spectrometer at 25 °C, and the resulting chemical shift perturbations were used to determine the K_D_ value as described above.

For the quantification of PIN1 isomerization of peptides IRAK-M-pS110 and IRAK-M- S110E, homonuclear 2D rotating-frame overhauser effect spectroscopy (ROESY) NMR experiments were performed. For PIN1 catalysis of IRAK-M-pS110, 13.8 µM of PIN1 was added to 4.44 mM of peptide, and ROESY experiments were acquired with 0 ms, 4 ms, 8 ms, 20 ms, 40 ms, 60 ms, 80 ms, 100 ms, and 150 ms mixing times. For PIN1 catalysis of IRAK-M-S110E, 20 µM of PIN1 was added to 4.18 mM of peptide, and ROESY experiments were acquired with 0 ms, 16 ms, 20 ms, 40 ms, 60 ms, 80 ms, 100 ms, and 150 ms mixing times. For the appropriate controls, each of IRAK-M-pS110 and IRAK-M-S110E were detected by ROESY without PIN1. The ratios *trans* to *cis* were measured by Total Correlation Spectroscopy (TOCSY) for both peptides. The intensity ratios of cross peaks to diagonal peaks for *cis* and *trans* conformation in the ROESY spectra were fit using Equations 1 – 6^65^:

1 $$I_{\rm cc}(t_{\rm m}) = \frac{{I_{\rm cc}(0)\{ - \left( {\lambda _2 - a_{11}} \right){\rm e}^{ - \lambda _1t_{\rm m}} + \left( {\lambda _1 - a_{11}} \right){\rm e}^{ - \lambda _2t_{\rm m}}\} }}{{\lambda _1 - \lambda _2}}$$

2 $$I_{\rm tt}(t_{\rm m}) = \frac{{I_{\rm tt}(0)\{ - \left( {\lambda _2 - a_{22}} \right){\rm e}^{ - \lambda _1t_{\rm m}} + \left( {\lambda _1 - a_{22}} \right){\rm e}^{ - \lambda _2t_{\rm m}}\} }}{{\lambda _1 - \lambda _2}}$$

3 $$I_{\rm ct}(t_{\rm m}) = \frac{{I_{\rm cc}(0)\{ a_{21}e^{ - \lambda _1t_{\rm m}} - a_{21}{\rm e}^{ - \lambda _2t_{\rm m}}\} }}{{\lambda _1 - \lambda _2}}$$

4 $$I_{\rm tc}(t_{\rm m}) = \frac{{I_{\rm tt}(0)\{ a_{12}{\rm e}^{ - \lambda _1t_{\rm m}} - a_{12}{\rm e}^{ - \lambda _2t_{\rm m}}\} }}{{\lambda _1 - \lambda _2}}$$

5 $$\lambda _{1,2} = 1/2\{ (a_{11} + a_{22}) \pm \sqrt {(a_{11} - a_{22})^2 + 4k_{\rm ct}^{\rm cat}k_{\rm tc}^{\rm cat}} \}$$

6 $$a_{11} = k_{\rm ct}^{\rm cat} + R_{2,{\rm c}},a_{22} = k_{\rm tc}^{\rm cat} + R_{2,{\rm t}},a_{12} = - k_{\rm tc}^{\rm cat},a_{21} = - k_{\rm ct}^{\rm cat}$$

where *R*_2,c_ and *R*_2,t_ are the transverse relaxation rates of magnetization in *cis* and *tran*s, *t*_m_ is the mixing time, *k*_ct_^cat^ and *k*_tc_^cat^ represent the exchange rates between *cis* and *trans*, and *I*_cc_(0) and *I*_tt_(0) are the diagonal peak intensities of the *cis* and *trans* at states at time *t*_m_=0.

Homology modeling of the IRAK-M death domain sequence was performed using Swiss-Model^66^. From the several potential template structures available, an IRAK-2 death domain subunit within the Myddosome assembly structure (3mop.pdb, Chain L) was chosen for modeling to provide insight regarding interaction interfaces. The resulting homology model was energy minimized, and then aligned with IRAK-2 subunits in the Myddosome structure (3mop.pdb) using DeepView^67^.

### Resonance assignments and structure determination

A ^15^N IRAK-M[1-119:R56D,Y61E] sample was used to collect 2D 15N-1H HSQC spectra and 3D ^1^H-^15^N NOESY and ^1^H-^15^N TOCSY spectra. A ^13^C/^15^N double labeled sample was used to collect ^1^H-^15^N HSQC, HNCA, HN(CO)CA, HNCO, HN(CA)CO, CBCANH, CBCA(CO)NH, H(CCO)NH, C(CO)NH, HCCH-TOCSY, ^1^H-^13^C aliphatic NOESY, and ^1^H-^13^C aromatic NOESY spectra. Backbone assignments were obtained from ^1^H-^15^N HSQC, ^1^H-^15^N NOESY, ^1^H-^15^N TOCSY, HNCA, HN(CO)CA, HNCO, HN(CA)CO, CBCANH, CBCA(CO)NH, H(CCO)NH, C(CO)NH, and HCCH-TOCSY spectra. Aromatic ring resonances were assigned using the ^1^H-^15^N NOESY, ^1^H-^13^C aliphatic NOESY, and ^1^H-^13^C aromatic NOESY spectra. Carbonyl resonances were assigned using the HNCO spectrum.

Resonance assignments were obtained using PINE imbedded in NMRFAM-SPARKY and were adjusted manually. The assigned residue numbers are five added to the original IRAK-M residue numbering due to the non-native 5 residues at the N-terminus that remain after cleaving MBP.

The resulting chemical shift assignments and NOESY data were used for solving the IRAK-M[1-119:R56D,Y61E] structure (RCSB Protein Data Bank ID 5UKE). Chemical shifts and three sets of raw NOESY spectra in ‘.ucsf’ format were submitted to Ponderosa Client (http://ponderosa.nmrfam.wisc.edu/), and Ponderosa Analyzer was used to interatively refine and validate the resulting structural ensemble. Ponderosa uses CYANA automation and XPLOR^65^-NIH^68–70^.

### Protein docking simulations

Docking simulations were performed using the protein docking prediction server ClusPro (https://cluspro.bu.edu/home.php). ClusPro can simulate protein docking of oligomer forms, which was essential for the studies performed here^40,41^. The mutated IRAK-M residues (R56D and Y61E) were restored in silico to their wild type identities using the Mutate tool in SwissPdbView. For docking of IRAK-M DD into a Myddosome containing IRAK1, SwissModel was used to build a homology model of the IRAK1 DD using IRAK2 DD subunits L, M and N in the 3MOP structure as templates, and three copies of the resulting homology model were aligned with the L, M and N IRAK2 subunits in the Myddosome complex (3MOP) to generate a Myddosome model with three IRAK1 DD subunits and an empty space at the K subunit position. IRAK-M was docked with this modified oligomer (composed of six MyD88, four IRAK4 and three IRAK1 DD subunits) using ClusPro. For docking of IRAK-M DD into a Myddosome containing IRAK2, the K subunit of IRAK2 was removed from the Myddosome structure, and docking of IRAK-M DD to this complex (composed of six MyD88, four IRAK4 and three IRAK2 DD subunits) was simulated using ClusPro.

### Patient study enrollment and segmental allergen challenge

For human subject study, non-smokers with a history of mild asthma with an FEV_1_ greater than 70% of predicted, using only intermittent beta-agonists for treatment, who were between the ages of 18 and 55 were recruited to undergo segmental allergen challenge via bronchoscopy. Participants were selected on the basis of both a positive methacholine PC20 < 8 mg/mL and a positive skin prick test to *Dermatophagoides pternyssinus* (DerP1). A positive intradermal that yielded a reaction at or below the concentration threshold of 0.1 AU/mL following the methods set forth in Parulekar et al., was also required, although only the DerP1 antigen was used.

All participants (Table 2) were enrolled at Brigham and Women’s Hospital (BWH) in Boston, MA and all procedures were performed at BWH. Institutional Review Board approval was obtained at the site and each participant provided written informed consent. The study was registered with ClinicalTrials.gov (NCT01691612). For further analysis the human samples were de-identified

**Table 2:** Demographic and clinical parameters for study participants

Patient number	Race	Age	Baseline FEV1	BAL Eosinophils (Pre-Challenge) %	BAL Eosinophils (Post-Challenge) %
1	Caucasian/Non-Hispanic	29	3.30 L/102.7%	0%	5%
2	Caucasian/Hispanic	21	3.44 L/89.6%	0.2%	76.1%
3	Black/Non-Hispanic	24	3.09 L/87.0%	0%	0.2%
4	Caucasian/Non-Hispanic	28	4.23 L/82.2%	0%	84.9%

### Inclusion criteria

- Patients 18-55 years of age, diagnosed with asthma for at least 1 year;
- And FEV1 >70% predicted on only short acting beta agonists
- And methacholine PC20 < 8 mg/ml
- Positive skin prick test to *Dermatophagoides pteronyssinus (*DerP)
- Positive reaction to a concentration of *Dermatophagoides pteronyssinus (*DerP) less than or equal to 1:100,000 dilution of a 10,000 AU/mL stock solution or 0.1 AU/mL during intradermal skin testing.
- No prior history of intubation for asthma
- No use of inhaled corticosteroids for 1 month prior to entry

### Exclusion criteria

- Current smoking or smoking history of greater than 10 pack-years
- Any other clinically important comorbidity determined by the principal investigator to affect subject safety, including uncontrolled diabetes, uncontrolled coronary artery disease, acute or chronic renal failure, and uncontrolled hypertension that would increase the risk of significant adverse events during bronchoscopy,
- Worsening of asthma symptoms requiring treatment with steroids within 4 weeks of screening
- Respiratory infection within four weeks
- Women of child-bearing potential, defined as all women physiologically capable of becoming pregnant or who are currently pregnant or lactating.

Unless they:

- Are women whose career, lifestyle, or sexual orientation precludes intercourse with a male partner
- Are women whose partners have been sterilized by vasectomy or other means
- Use one acceptable birth control method. Adequate barrier methods of contraception include: diaphragm, condom (by the partner), intrauterine device (copper or hormonal), sponge or spermicide. Hormonal contraceptives include any marketed contraceptive agent that includes an estrogen and/or a progestational agent.

- Pre-existing lung disease other than asthma
- History of coagulation disorders or abnormal PT/PTT testing at screening
- History of immunodeficiency diseases, including HIV
- A disability that may prevent the patient from completing all study requirements
- Use of other investigational drugs at the time of enrollment, or within 30 days or 5 half-lives of enrollment, whichever is longer
- History of malignancy of any organ system (other than localized basal cell carcinoma of the skin), treated or untreated, within the past 5 years, regardless of whether there is evidence of local recurrence or metastases.
- Diagnosis of Hepatitis B or C.
- History of alcohol abuse (as determined by the principal investigator) within 6 months of screening.
- History of illicit drug abuse (as determined by the principal investigator) within 6 months of screening.

### Segmental allergen challenge and first bronchoscopy

After recoding baseline vitals, PO_2_, FEV1, PEFR, appropriate anesthetic medication was administered. Then, a fiberoptic bronchoscope with bronchoalveolar lavage (BAL) was performed by sequential instillation and removal of 5 aliquots of 50 mL normal saline in the lingula. Brushings and biopsies were taken after the BAL. The bronchoscope was then re-wedged on the contra lateral side (right middle lobe) in a readily identifiable sub-segment. A safety dose (100 × the minimum dose that caused reaction during the intradermal) of 5 mL of allergen in a prefilled syringe was instilled followed by 5 mL of air and the wedge was maintained for 5 minutes. Subject’s safety parameters were assessed after administration of the sub-threshold allergen dose. If the investigator determined the subject had well tolerated the safety dose, full dose of allergen (1000 × the minimum dose that caused reaction during the intradermal) in a pre-filled syringe was administered followed by 5 ml of air and the wedge was maintained for 5 min. Antigen lot to lot consistency was maintained between the participants intradermal skin test and segmental challenge. Participants were monitored for safety until 2 h after the procedure. After allergen challenge, if the subject developed systemic allergic reaction such as diffuse urticaria, angioedema, stridor, hypotension, syncope or any other serious adverse event, subject would be dropped from the study and only be followed up for safety reasons. Participants were not be allowed to leave for home until their condition was stable as assessed by the study physician.

### Second bronchoscopy

Pre-bronchoscopy and bronchoscopy eligibility procedures were followed as detailed above prior to 2^nd^ bronchoscopy. Specimens taken were BAL first, followed by brushings, and finally biopsies. These were taken where the segmental allergen challenge was placed (right middle lobe). Drug and post-procedure follow up and monitoring were followed as detailed above.

### Statistical analysis

Experiments were routinely repeated at least three times, and the repeat number was increased according to effect size or sample variation. We estimated the sample size considering the variation and mean of the samples. No statistical method was used to predetermine sample size. No animals or samples were excluded from any analysis. Animals were randomly assigned groups for in vivo studies; no formal randomization method was applied when assigning animals for treatment. Group allocation and outcome assessment was not done in a blinded manner, including for animal studies. Values are reported as means ± standard errors of the means (SEM) unless otherwise stated; the data were analyzed by a Student’s two-tailed *t* test or with Mann-Whitney U test. The significance set at a *P* value of <0.05 or highly significance set at a *P* value of < 0.01.

### Data availability

The authors declare that the data supporting the findings of this study are available within the article and its Supplementary Information files, or are available upon reasonable requests to the authors. The Gene array data discussed in this publication have been deposited in NCBI’s Gene Expression Omnibus, and are accessible under the accession code GSE66431. The ^1^H-^15^N HSQC spectrum of ^15^N-IRAK-M [1–119:R56D,Y61E] as depicted by the NMR are deposited at the BioMagResBank (http://www.bmrb.wisc.edu/), and are accessible under the accession code 30237 or directly via http://www.bmrb.wisc.edu/data_library/summary/index.php?bmrbId=30237. The resulting NMR structure was deposited in the RCSB Protein Data Bank (PDB ID 5UKE).

## Electronic supplementary material

Supplementary Information
