# Author Correction: The IL-33-PIN1-IRAK-M axis is critical for type 2 immunity in IL-33-induced allergic airway inflammation

**DOI:** 10.1038/s41467-023-39297-5

**Published:** 2023-06-19

**Authors:** Morris Nechama, Jeahoo Kwon, Shuo Wei, Adrian Tun-Kyi, Robert S. Welner, Iddo Z. Ben-Dov, Mohamed S. Arredouani, John M. Asara, Chun-Hau Chen, Cheng-Yu Tsai, Kyle F. Nelson, Koichi S. Kobayashi, Elliot Israel, Xiao Zhen Zhou, Linda K. Nicholson, Kun Ping Lu

**Affiliations:** 1 Division of Translational Therapeutics, Department of Medicine and Cancer Research Institute, Beth Israel Deaconess Medical Center, Harvard Medical School, Boston, MA 02215 USA; 2 Department of Molecular Biology & Genetics, Cornell University, Ithaca, NY 14853 USA; 3 Department of Nephrology and Hypertension, Hadassah-Hebrew Medical Center, 91120 Jerusalem, Israel; 4 Department of Medicine, Brigham and Women’s Hospital, Boston, MA 02115 USA; 5 Department of Microbial Pathogenesis & Immunology, Texas A&M Health Science Center, College Station, TX 77843 USA; 6 Broad Institute of MIT and Harvard, Cambridge, MA 02142 USA; 7 Institute for Translational Medicine, Fujian Key Laboratory for Translational Research in Cancer and Neurodegenerative Diseases, Fujian Medical University, Fuzhou, 350108 Fujian China

Correction to: *Nature Communications* 10.1038/s41467-018-03886-6, published online 23 April 2018

In this article, the author name Adrian Tun-Kyi was incorrectly written as Adrian Tun Kyi and the given and family names were incorrectly structured. The original article has been corrected.

