# Supplementary material for: The IL-33-PIN1-IRAK-M axis is critical for type 2 immunity in IL-33-induced allergic airway inflammation
Source: Nat Commun. 2018 Apr 23;9:1603. doi: 10.1038/s41467-018-03886-6 (PMC5913134; doi:10.1038/s41467-018-03886-6)
Supplement: Supplementary file 1 — Supplementary Information [file 41467_2018_3886_MOESM1_ESM.pdf]

Supplementary information for:

The IL-33-PIN1-IRAK-M axis is critical for type 2 immunity in IL-33-induced allergic airway inflammation

Morris Nechama, Jeahoo Kwon, Shuo Wei, Adrian Tun Kyi, Robert S. Welner, Iddo Z. Ben-Dov, Mohamed S. Arredouani, John M. Asara, Chun-Hau Chen, Cheng-Yu Tsai, Kyle F. Nelson, Koichi S Kobayashi, Elliot Israel, Xiao Zhen Zhou, Linda K. Nicholson and Kun Ping Lu.

Supplementary Figure 1.

a

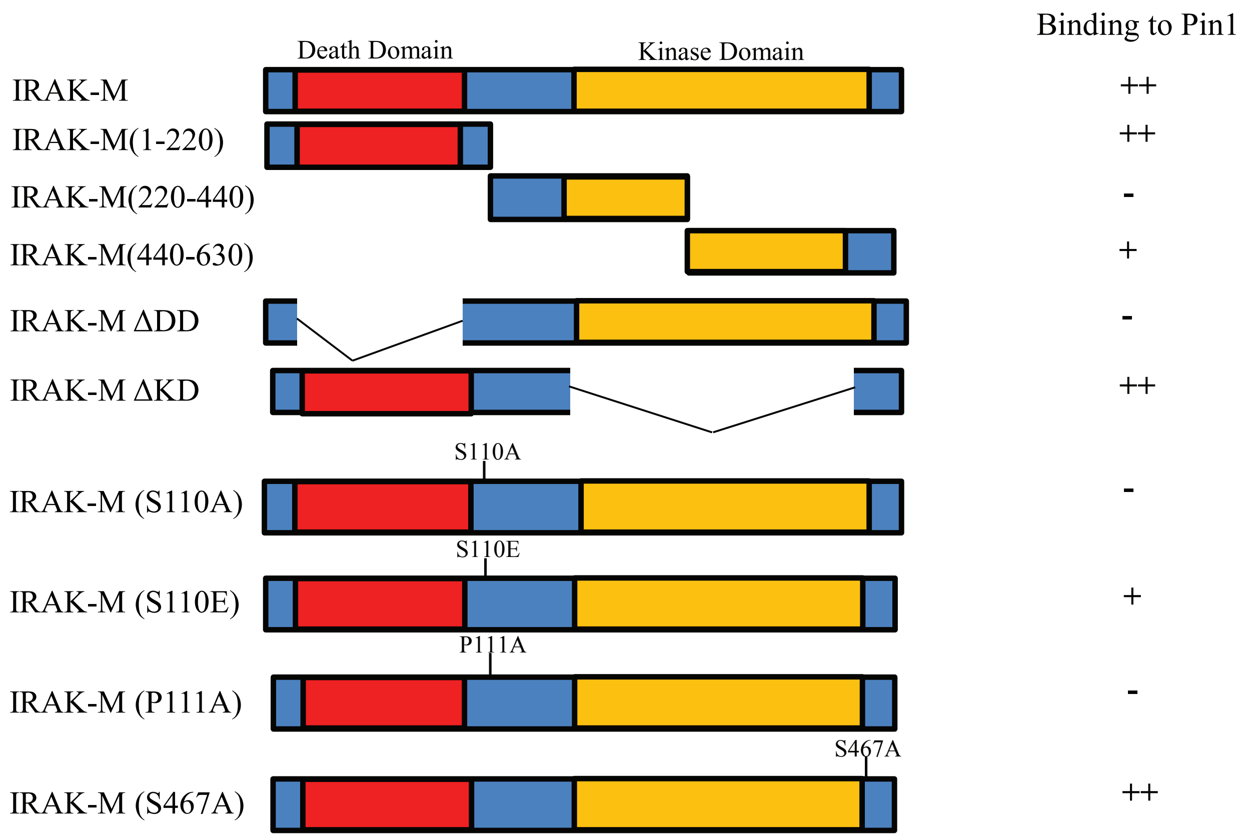

b

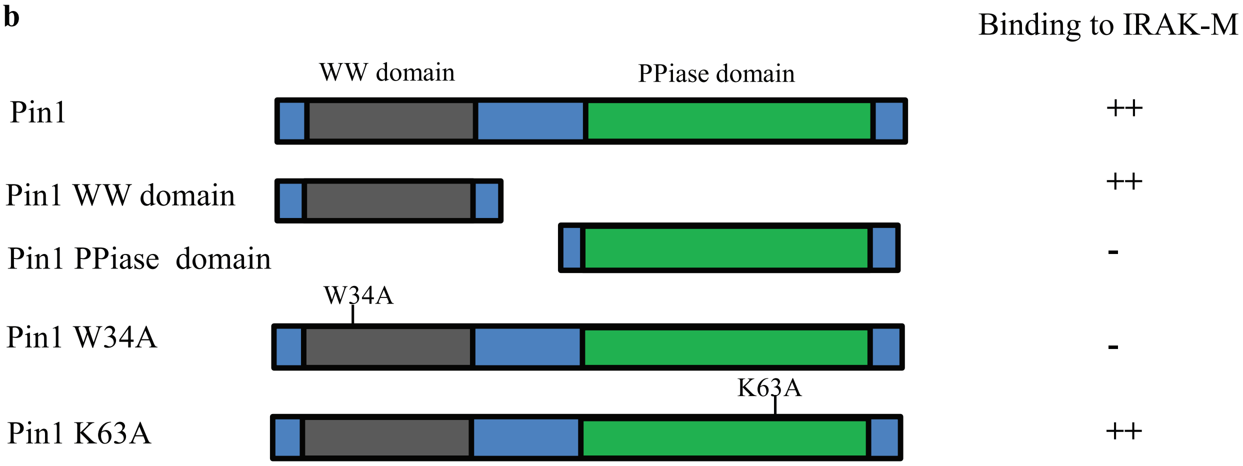

Supplementary Figure 1. Summarization of Pin1 and IRAK-M interaction. (**a**) Diagram showing IRAK-M and its mutant and their capability to interact with Pin1. (**b**) Diagram showing Pin1 and its mutant and their capability to interact with IRAK-M

Supplementary Figure 2.

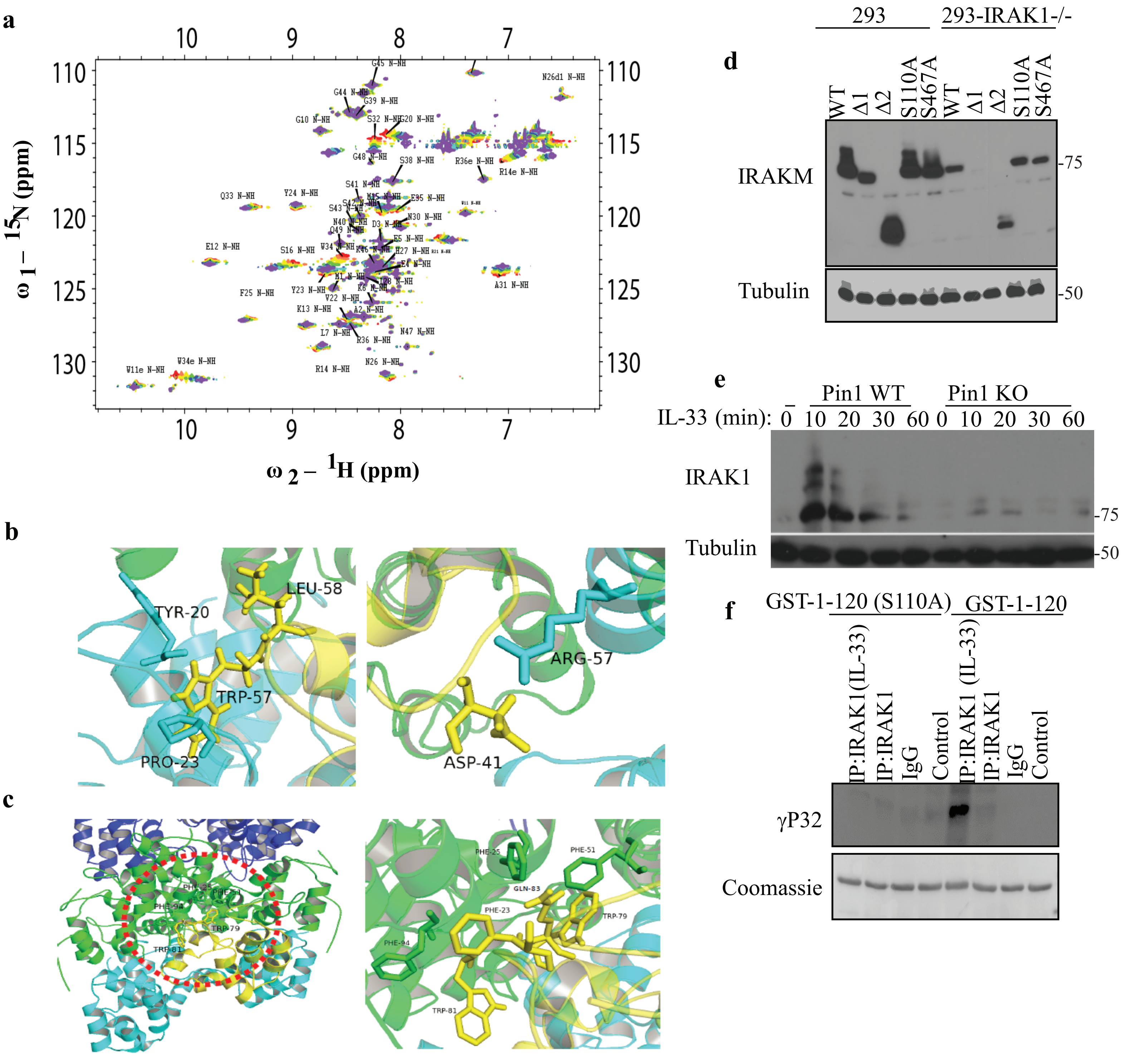

Supplementary Figure 2. IRAK-M DD structure modeling analysis and phosphorylation by IRAK1. (a) Overlaid 1H-15N HSQC spectra of the 15N-labeled Pin1 WW domain showing changes in chemical environment resulting from titration with the phosphorylated IRAK-M peptide. Overlaid spectra of apo (red) and increasing amounts of ligand (rainbow of colors, with purple as highest ligand concentration) show progressive peak shifts. (b) Interaction between IRAK-M DD and IRAK1 DD from Figure 3f. Cyan: IRAK1 DDs, Yellow: IRAK-M DD. (c) The aromatic residues interaction between IRAK-M DD and IRAK4 DD from Figure 3f. Green: IRAK4 DDs, Yellow: IRAK-M DD. (d) IRAK-M or its mutants as described, were expressed in 293 cells or 293 IRAK1null cells expressing the ST2 receptor and treated with IL-33. The phosphorylation is shown by the slow migrating bands. (e) WT or Pin KO cells were treated with IL-33 and IRAK1 phosphorylation was monitored at different time after stimulation. (f) DC2.4 cells were either treated or not with IL-33. IRAK1 was IP'd and incubated with GST-IRAK-M aa1-120 in the presence of ΓP32ATP. The beads were washed and GST-IRAK-M was eluted and run for autoradiography.

Supplementary Figure 3.

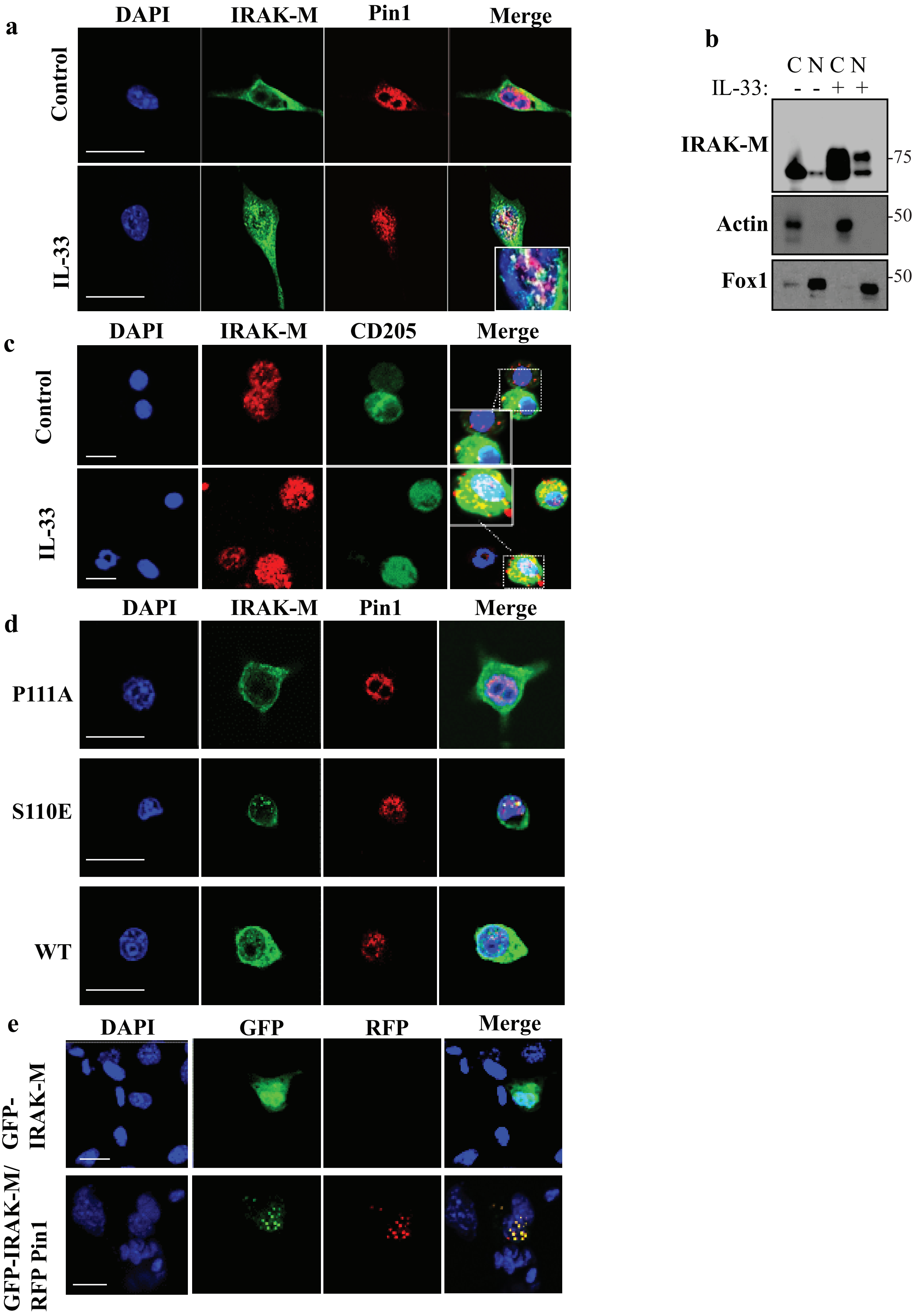

Supplementary Figure 3. Pin1 targets IRAK-M and promotes its nuclear translocation upon IL-33 induction. (**a**) Immunostaining for IRAK-M and Pin1 in DC2.4 cells before and after IL-33 treatment. (**b**) DC2.4 cells were treated with IL-33 and subjected to nuclear/cytoplasmic fractionation, followed by Western blot for IRAK-M with actin as a cytoplasmic marker and Fox1 as a nuclear marker. C- cytoplasmic; N- Nuclear. (**c**) Cytospin slides from BALF of the treated WT mice as indicated, were co stained for DC205 and IRAK-M. (**d**) IRAK-M or its mutants S110E or P111A were stably expressed in DC2.4 cells and their localization and

Supplementary Figure 4.

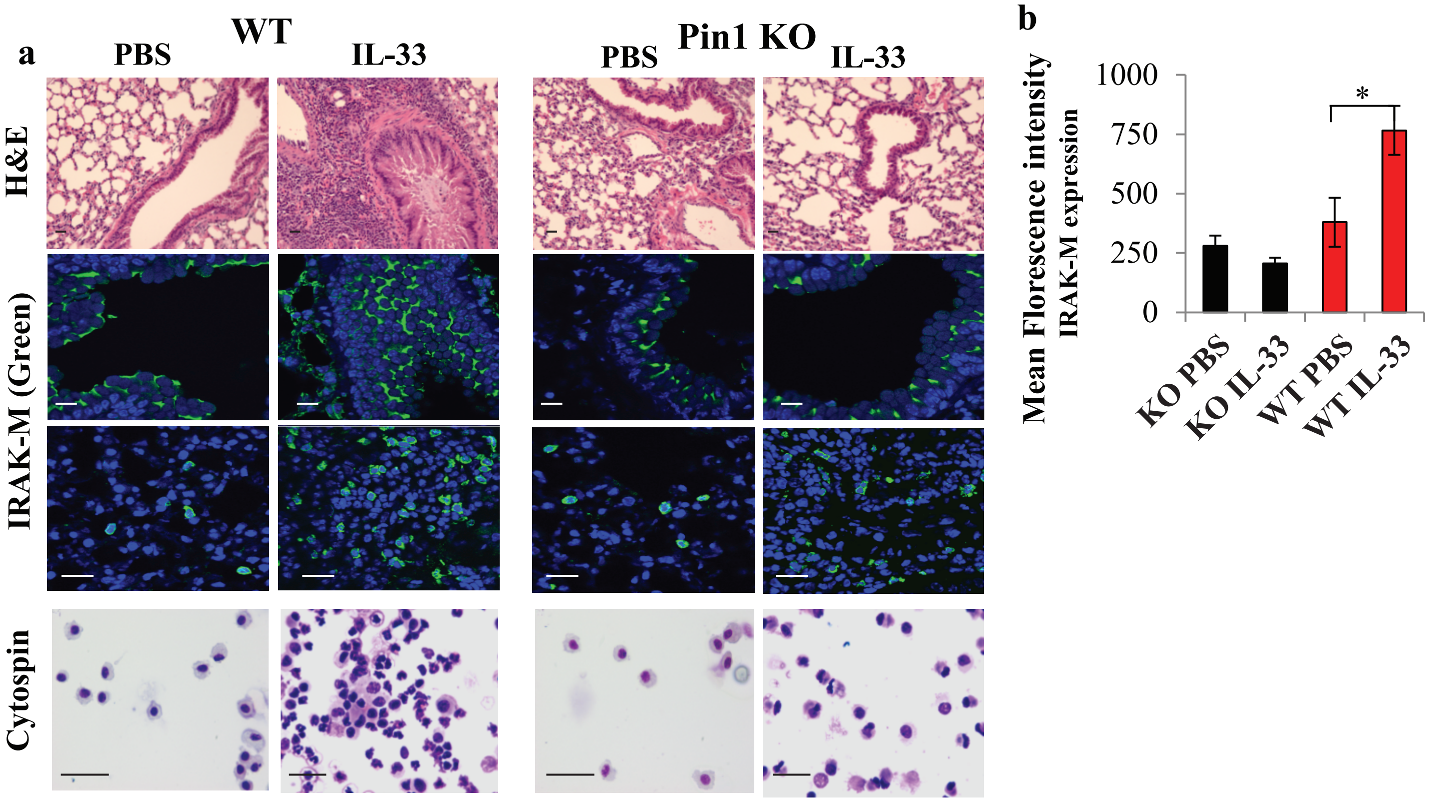

Supplementary Figure 4. IRAK-M is necessary for IL-33 induce inflammation.
(**a**) Representative H&E staining and immune fluorescence staining for IRAK-M and BALF cytospin cells in lung sections of WT and Pin1 KO mice treated with PBS or IL-33, (n=3). Scale bar = 50 µm. (**b**) Mean fluorescence intensity quantification of IRAK-M positive immune infiltrating cells using Velocity program software (n=3). The data were analyzed by a Student's two-tailed t test and the values are reported as mean ± standard errors of the means (SEM). *- statistical significance (P<0.05), **- significance (P<0.01).

Supplementary Figure 5.

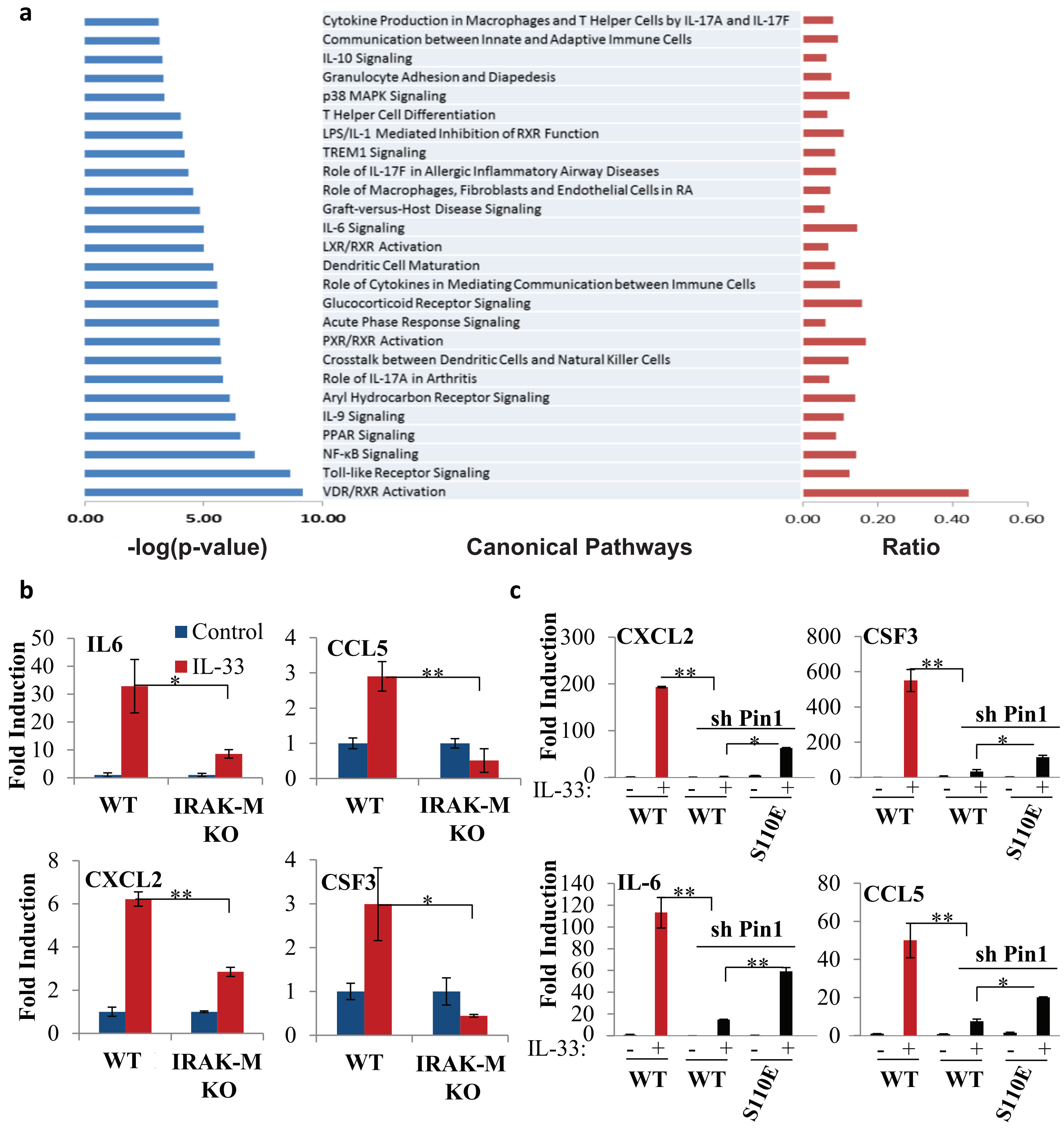

Supplementary Figure 5. IRAK-M is necessary for the expression of IL-6, CXCL2, CSF3 and CCL5 upon IL-33 induction. (**a**) Comparison of Top Signaling Pathways affected by IL-33 treatment in IRAK-M-over-expressing DC2.4 cells. Sets of differentially expressed genes (FC of 2) between IL-33- and PBS-treated cell lines were uploaded onto Ingenuity Pathway Analysis and corresponding signaling pathways predicted. Statistical significance was set at –log P=2. (**b**) WT BMDC and IRAK-M KO BMDC were either treated or not with IL-33 as before and the gene expression of IL-6, CXCL2, CSF3 and CCL5 were monitored by qRT–PCR. The levels are normalized to actin mRNA. (c) IRAK-M or its S110E mutant were stably expressed in Pin1 KD DC2.4 cells. The cells were treated with IL-33 for 24 hours before gene expression of IL-6, CXCL2, CSF3 and CCL5 were monitored by qRT-PCR. The data were analyzed by a Student's two-tailed t test and the values are reported as mean ± standard errors of the means (SEM). *- statistical significance (P<0.05), **- significance (P<0.01).

Supplementary Figure 6.

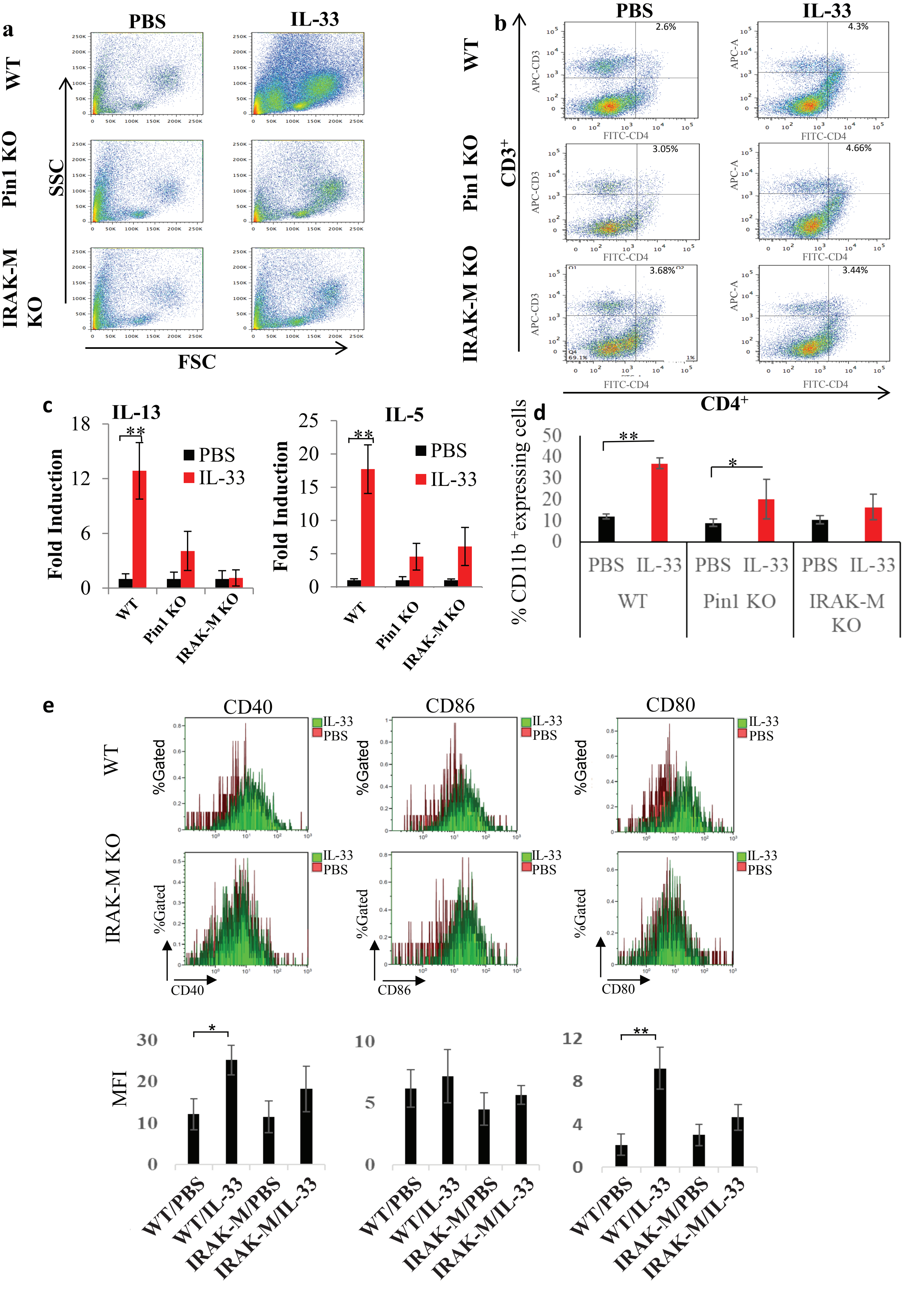

Supplementary Figure 6. CD3+ CD4+ T cell population in the lungs of the indicated mice after PBS or IL-33 challenge. (**a**) SSC/FSC of the indicated digested lungs. (**b**) The indicated lungs were digested and the CD3+ CD4+ cells were monitored and cell sorted using APC-CD3 and FITC-CD4 antibodies respectively, for further analysis. Representative of 3 different mice in each group. (**c**) Sorted CD3+ CD4+ cells from the indicated mice after PBS and IL-33 challenge were analyzed for IL-13 and IL-5 expression (n=3). (**d**) Quantification of figure 6E; Dendritic cell CD11b+ expression in WT, Pin1KO and IRAK-M KO mice lungs, either treated with IL-33 or PBS as before. (**e**) WT and IRAK-M KO mice were either treated with IL-33 or PBS, the lungs were digested and the CD11c+ cells were analyzed for the expression of costimulatory proteins CD40, CD80 and CD86. The representative of FACS plot and mean florescence intensity analysis is present (n=3). The data were analyzed by a Student's two-tailed t test and the values are reported as mean ± standard errors of the means (SEM). *- statistical significance (P<0.05), **- significance (P<0.01).

Supplementary Figure 7.

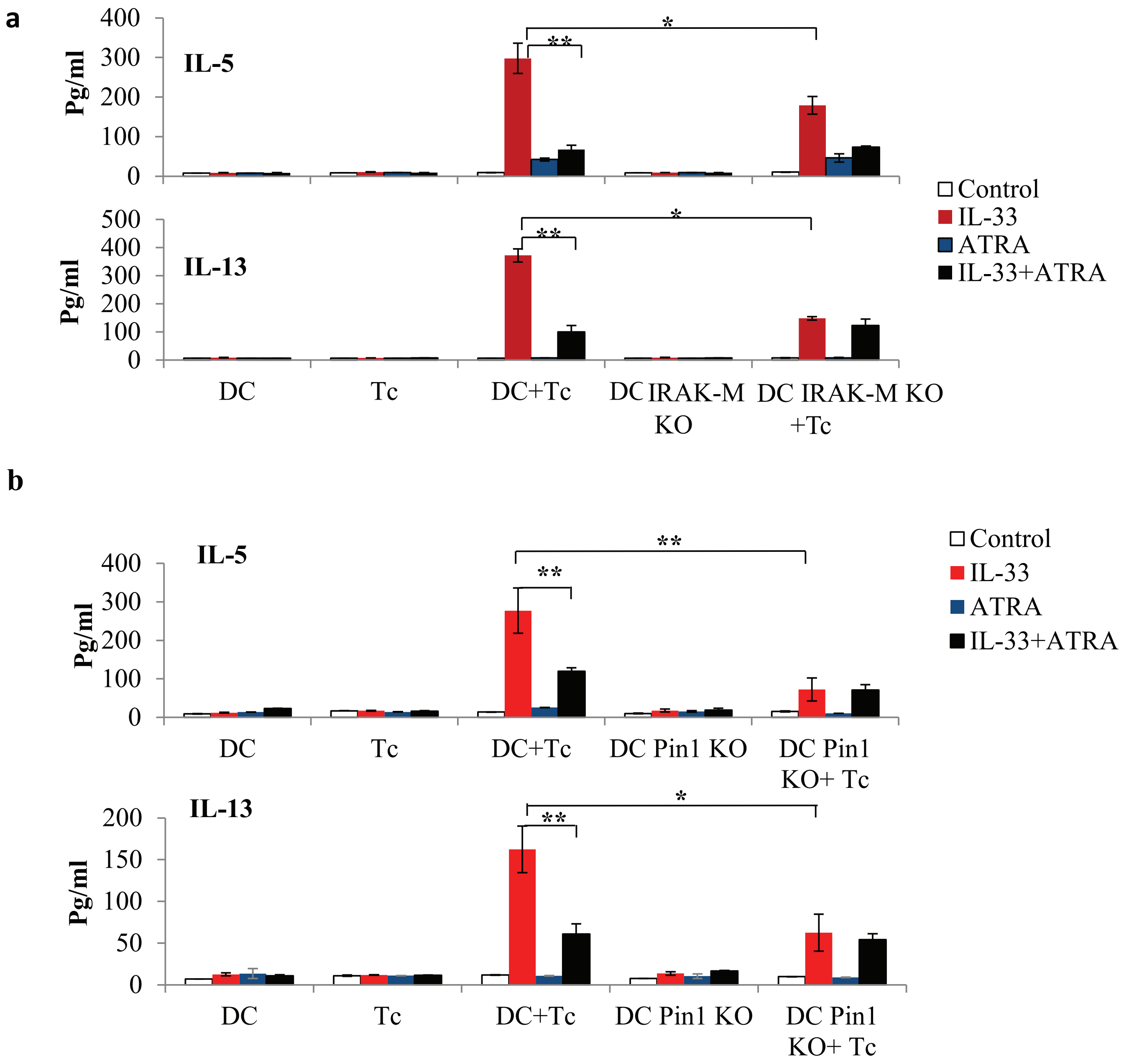

Supplementary Figure 7. IRAK-M KO or Pin1 KO derived bone marrow dendritic cells attenuates Naïve TH2 cell differentiation upon IL-33 induction in co culture experiment. Naïve CD4+ T cells isolated from C57 B6 mice were cultured with or without IL-33 (50ng/ml) and BMDCs isolated from WT, IRAK-M KO (**a**) or Pin1 KO mice (**b**) in a 1:5 ratio for 5 days with no antigen being added. In some cases as indicated 5 μM of ATRA was added to the medium two days prior to T cell co culturing and during the experiment. Supernatants were analyzed for cytokines on day 5. The data were analyzed by a Student's two-tailed t test and the values are reported as mean ± standard errors of the means (SEM). *- statistical significance ($P<0.05$), **- significance ($P<0.01$).

Supplementary Figure 8.

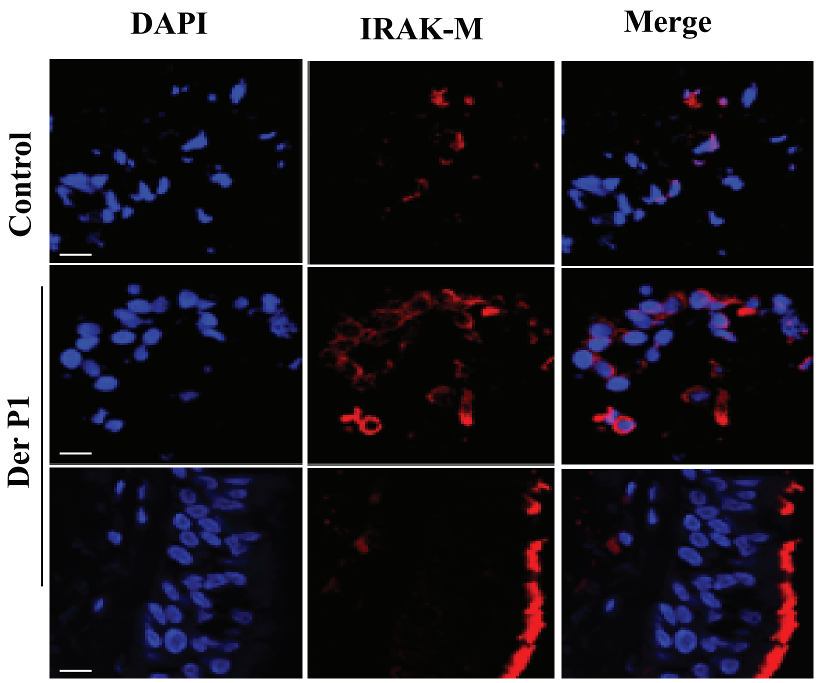

Supplementary Figure 8. Representative paraffin sections of biopsy samples from human patients, before and after Derp1 challenge were immune stained for IRAK-M.
Scale bar = 50 µm.

Figure 1a

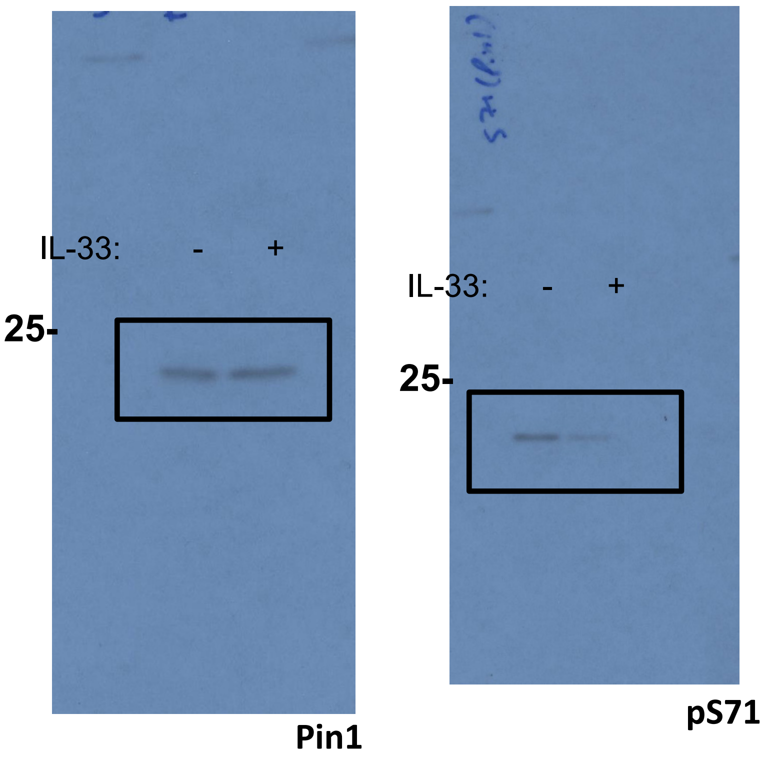

Supplementary Figure 9: Uncropped western blot images for figure 1a. Molecular weight (kDa) of the protein bands are indicated on the left side of the panels. Antibodies used to probe the nitrocellulose membranes are indicated on the bottom right. Rectangles represent the cropped images shown in figures. The cell treatment is indicated above the rectangles

Figure 2a

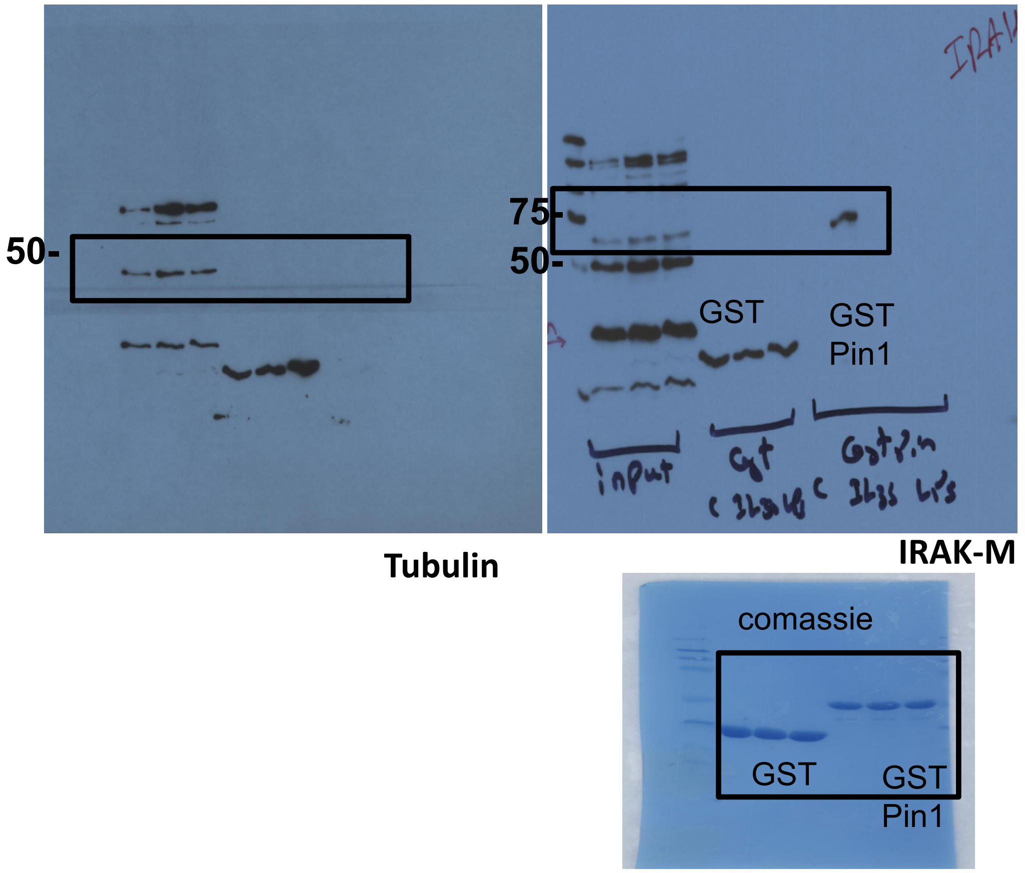

Supplementary Figure 10: Uncropped western blot images for figure 2a. Molecular weight (kDa) of the protein bands are indicated on the left side of the panels. Antibodies used to probe the nitrocellulose membranes are indicated on the bottom right. Rectangles represent the cropped images shown in figures. The Coomasie gel stain representing the GST and GST-PIN1 protein are indicated in the lower panel gel.

Figure 2b

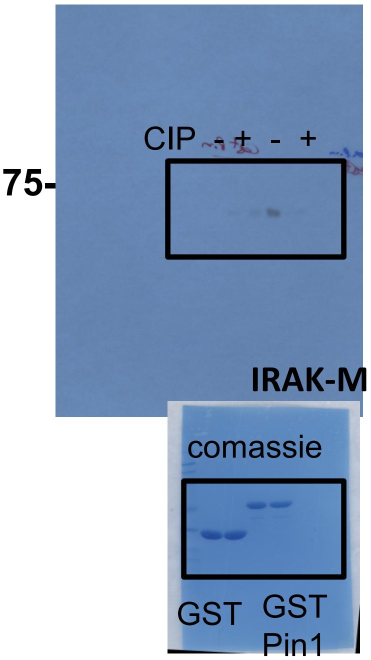

Figure 2c

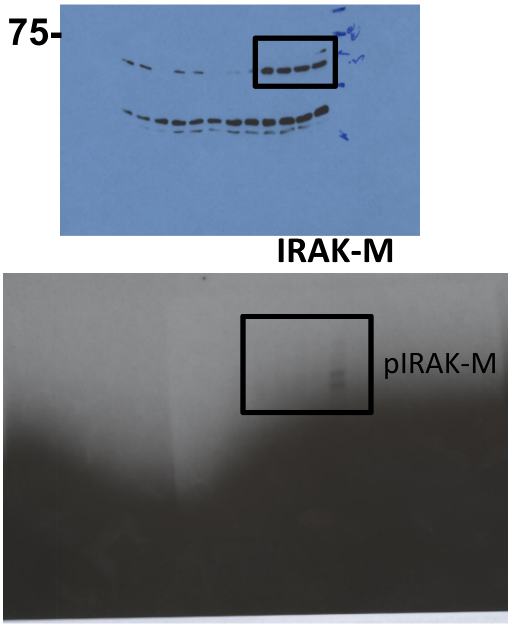

Supplementary Figure 11: Uncropped western blot images for figure 2b-c.(**2b**) Molecular weight (kDa) of the protein bands are indicated on the left side of the panels. Antibodies used to probe the nitrocellulose membranes are indicated on the bottom right. Rectangles represent the cropped images shown in figures. The Coomasie gel stain representing the GST and GST-PIN1 protein are indicated in the lower panel gel. The extract treatment indicated in the top of the rectangle. (**2c**) Molecular weight (kDa) of the protein bands are indicated on the left side of the panels. Antibodies used to probe the nitrocellulose membranes are indicated on the bottom right. Rectangles represent the cropped images shown in figures. In the lower panel the autoradiography blot is presented.

Figure 2d

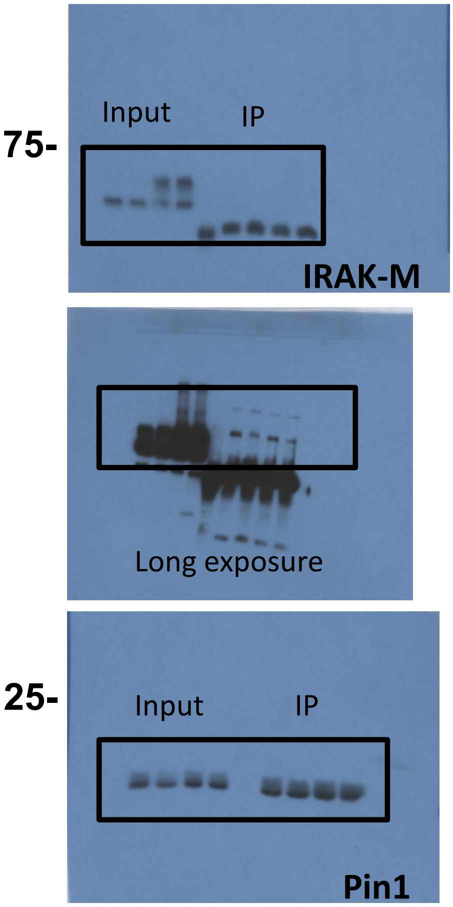

Figure 2e

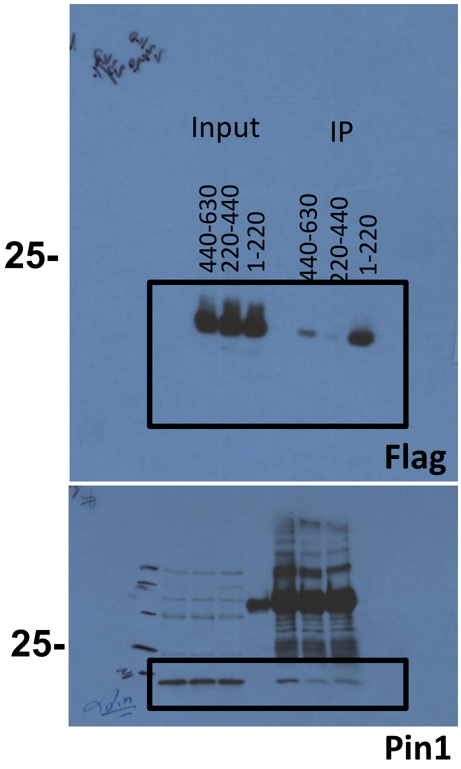

Supplementary Figure 12: Uncropped western blot images for figure 2d-e. (**2d**) Molecular weight (kDa) of the protein bands are indicated on the left side of the panels. Antibodies used to probe the nitrocellulose membranes are indicated on the bottom right. Rectangles represent the cropped images shown in figures. IP or Input extract is indicated. The mid blot represent longer exposure of the above gel. (**2c**) Molecular weight (kDa) of the protein bands are indicated on the left side of the panels. Antibodies used to probe the nitrocellulose membranes are indicated on the bottom right. Rectangles represent the cropped images shown in figures. IP or Input extract is indicated.

Figure 2f

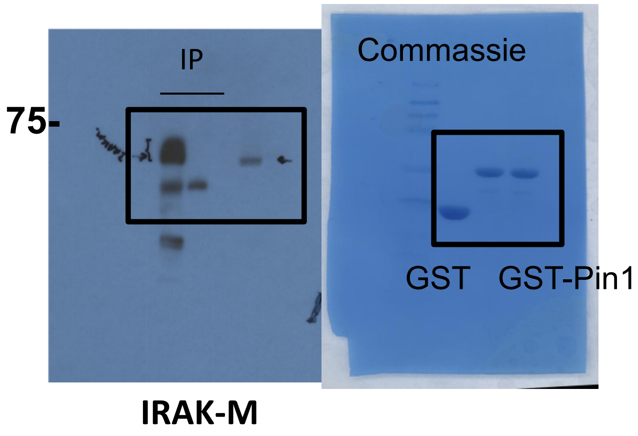

Supplementary Figure 13: Uncropped western blot images for figure 2f. Molecular weight (kDa) of the protein bands are indicated on the left side of the panels. Antibodies used to probe the nitrocellulose membranes are indicated on the bottom right. Rectangles represent the cropped images shown in figures. The Coomasie gel stain representing the GST and GST-PIN1 protein are indicated in the lower panel gel. The IP band is indicated on the top of the blot.

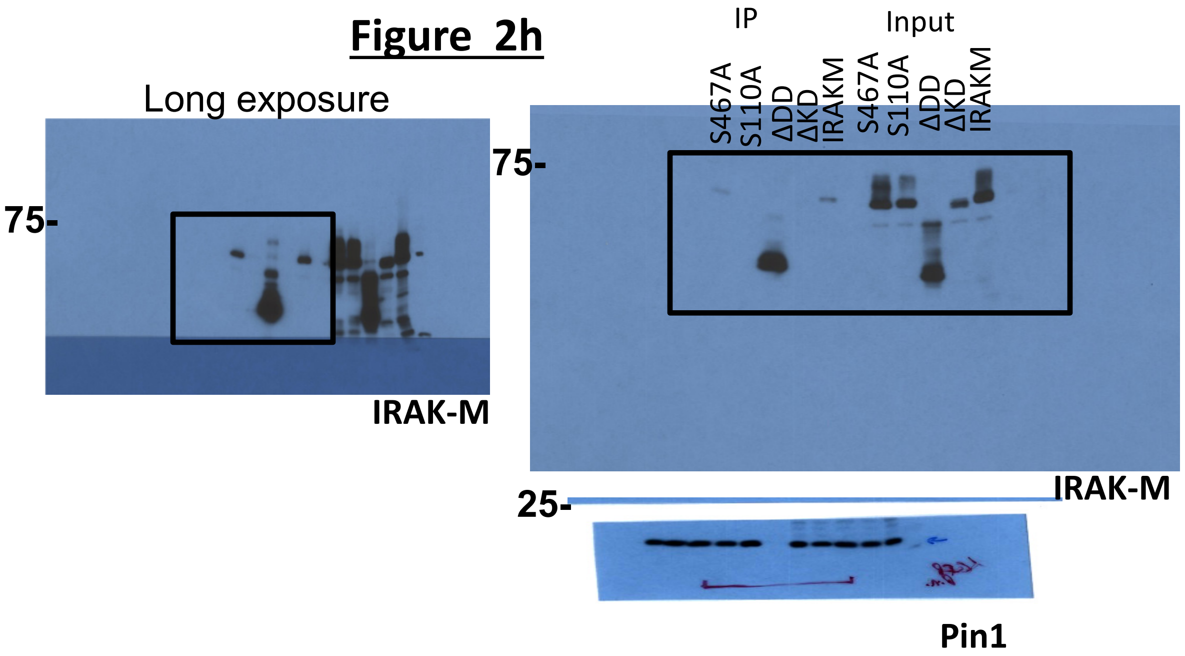

Supplementary Figure 14: Uncropped western blot images for figure 2h. Molecular weight (kDa) of the protein bands are indicated on the left side of the panels. Antibodies used to probe the nitrocellulose membranes are indicated on the bottom right. Rectangles represent the cropped images shown in figures. The IP and input extracts are indicated on the top of the blot. The different IRAK-M mutant used in this experiment are indicated.

Figure 2i

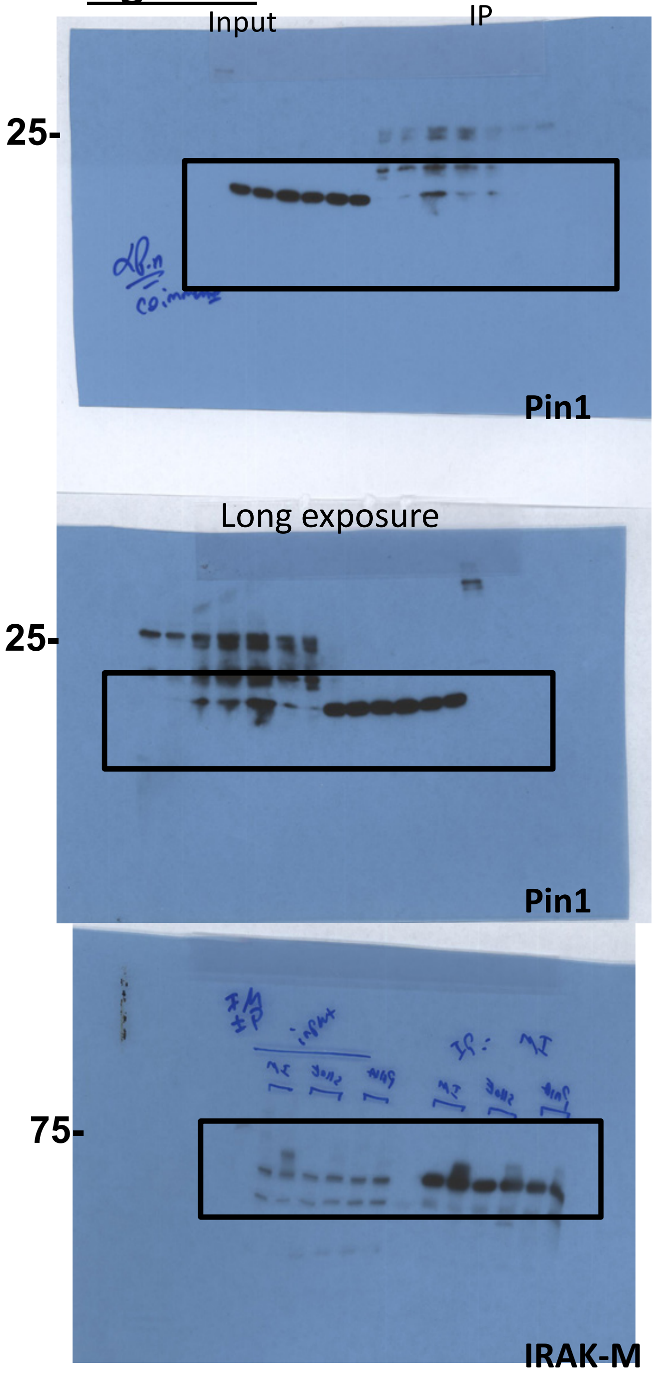

Supplementary Figure 15: Uncropped western blot images for figure 2i. Molecular weight (kDa) of the protein bands are indicated on the left side of the panels. Antibodies used to probe the nitrocellulose membranes are indicated on the bottom right. Rectangles represent the cropped images shown in figures. The IP and input extracts are indicated on the top of the blot. The mid blot represent longer exposure of the upper blot.

Figure 2j

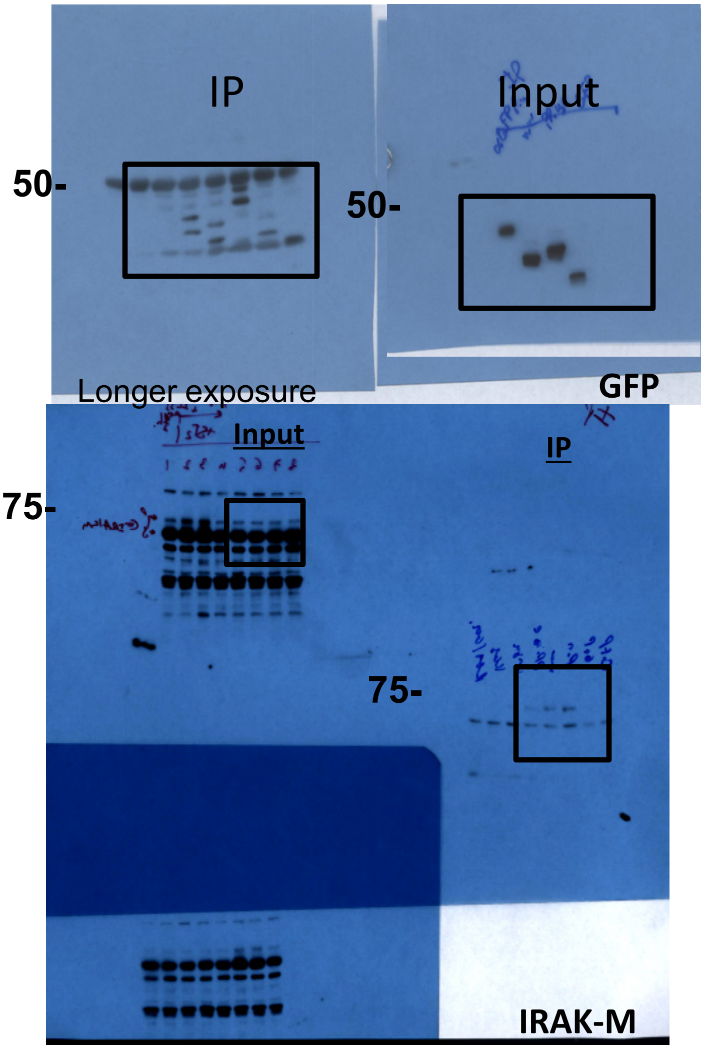

Figure 2k

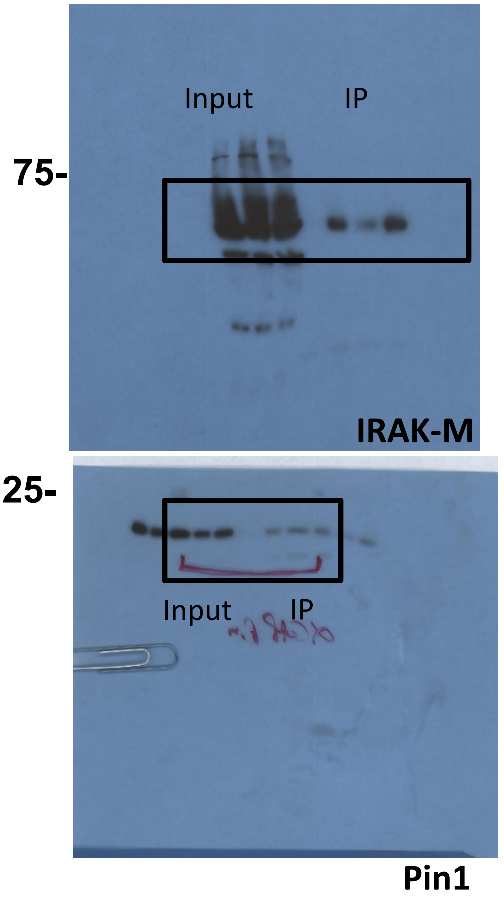

Supplementary Figure 16: Uncropped western blot images for figure 2j-k. (**2j**) Molecular weight (kDa) of the protein bands are indicated on the left side of the panels. Antibodies used to probe the nitrocellulose membranes are indicated on the bottom right. Rectangles represent the cropped images shown in figures. The IP and input extracts are indicated on the top of the blot. The blot representing longer exposure is indicated. (**2k**) Molecular weight (kDa) of the protein bands are indicated on the left side of the panels. Antibodies used to probe the nitrocellulose membranes are indicated on the bottom right. Rectangles represent the cropped images shown in figures. The IP and input extracts are indicated on the top of the blot.

Figure 4a

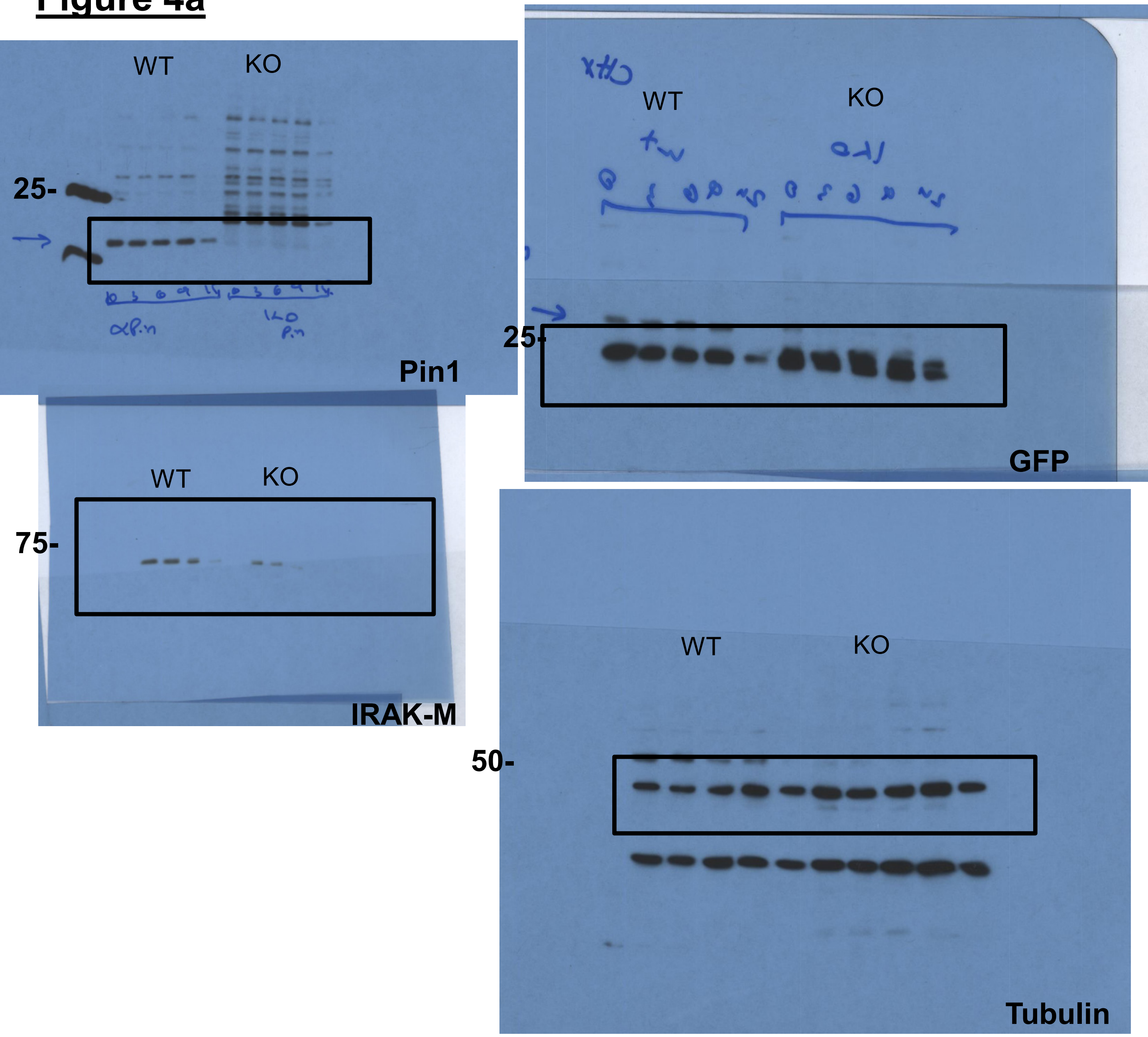

Supplementary Figure 17: Uncropped western blot images for figure 4a. Molecular weight (kDa) of the protein bands are indicated on the left side of the panels. Antibodies used to probe the nitrocellulose membranes are indicated on the bottom right. Rectangles represent the cropped images shown in figures. The WT and KO extracts

Figure 4c

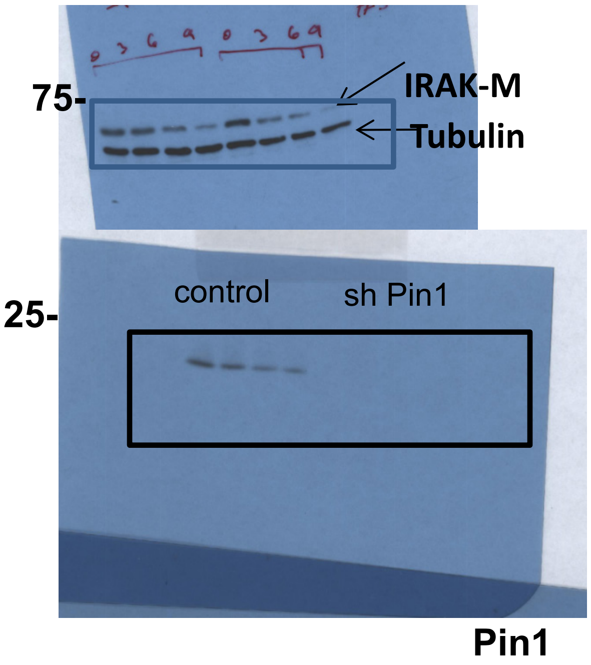

Figure 4e

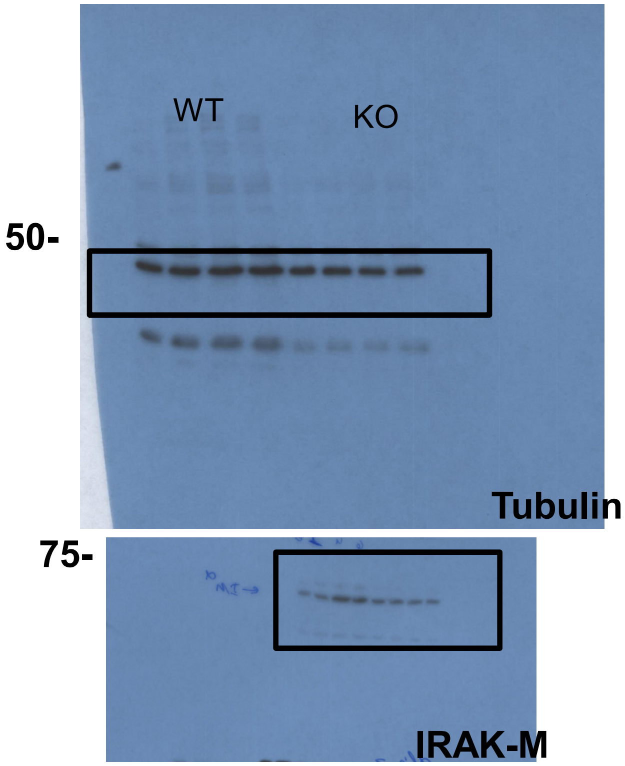

Supplementary Figure 18: Uncropped western blot images for figure 4c,e. (**4c**) Molecular weight (kDa) of the protein bands are indicated on the left side of the panels. Antibodies used to probe the nitrocellulose membranes are indicated on the bottom right. In the upper blot the antibodies are indicated with arrows. Rectangles represent the cropped images shown in figures. The control and shPin1 extracts are indicated. (**4e**)) Molecular weight (kDa) of the protein bands are indicated on the left side of the panels. Antibodies used to probe the nitrocellulose membranes are indicated on the bottom right. Rectangles represent the cropped images shown in figures. The WT and KO extracts are indicated.

Figure 4g

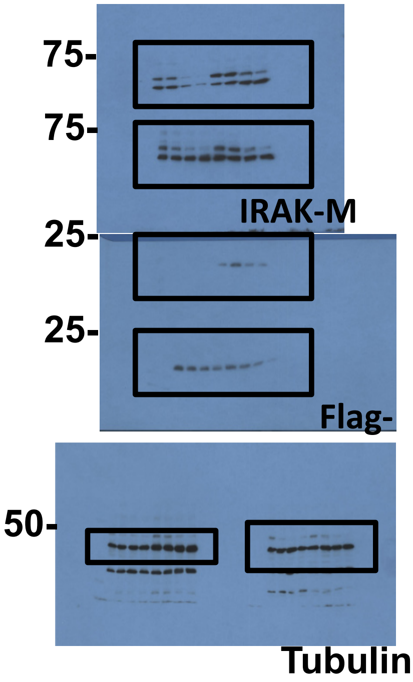

Figure 4i

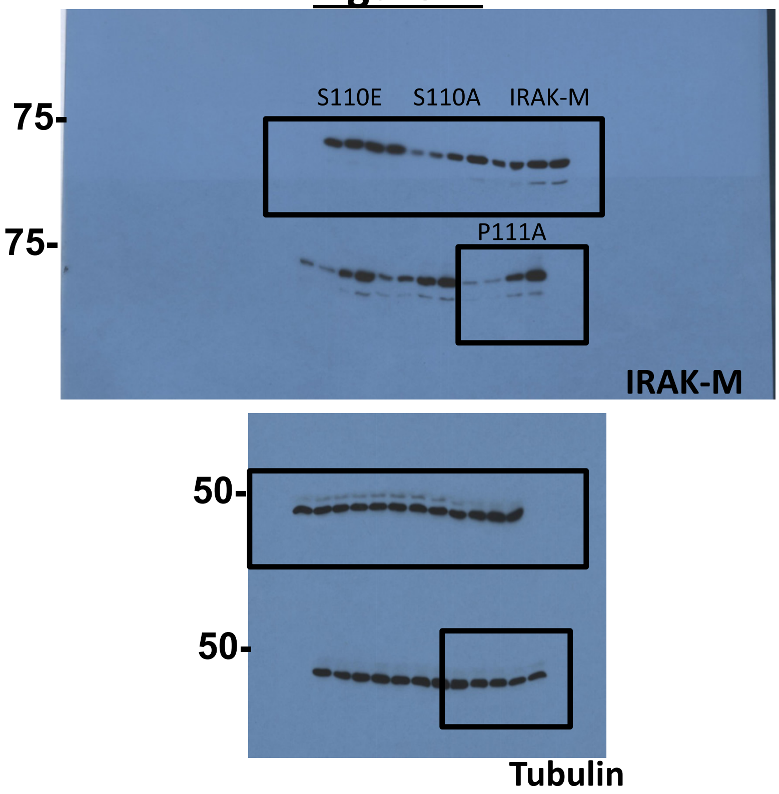

Supplementary Figure 19: Uncropped western blot images for figure 4g,i. (**4g**) Molecular weight (kDa) of the protein bands are indicated on the left side of the panels. Antibodies used to probe the nitrocellulose membranes are indicated on the bottom right. Rectangles represent the cropped images shown in figures. (**4i**) Molecular weight (kDa) of the protein bands are indicated on the left side of the panels. Antibodies used to probe the nitrocellulose membranes are indicated on the bottom right. Rectangles represent the cropped images shown in figures. The expression of IRAK-M and its different mutants are indicated.

Figure 4k

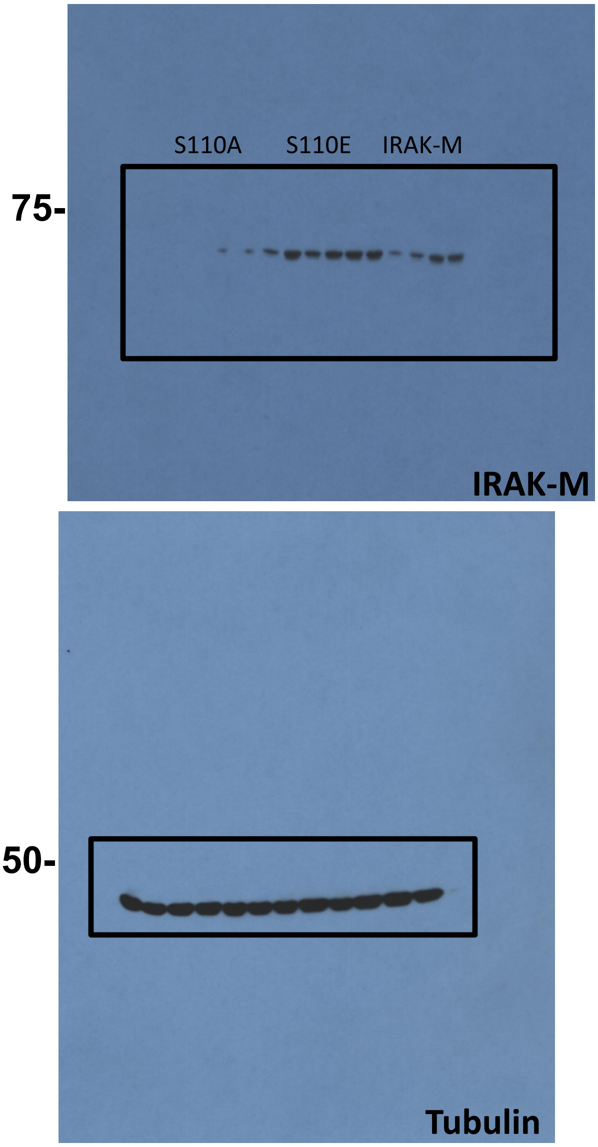

Supplementary Figure 20: Uncropped western blot images for figure 4k. Molecular weight (kDa) of the protein bands are indicated on the left side of the panels. Antibodies used to probe the nitrocellulose membranes are indicated on the bottom right. Rectangles represent the cropped images shown in figures. IRAK-M and its different mutants are indicated on the top of the blot.

Figure 5d

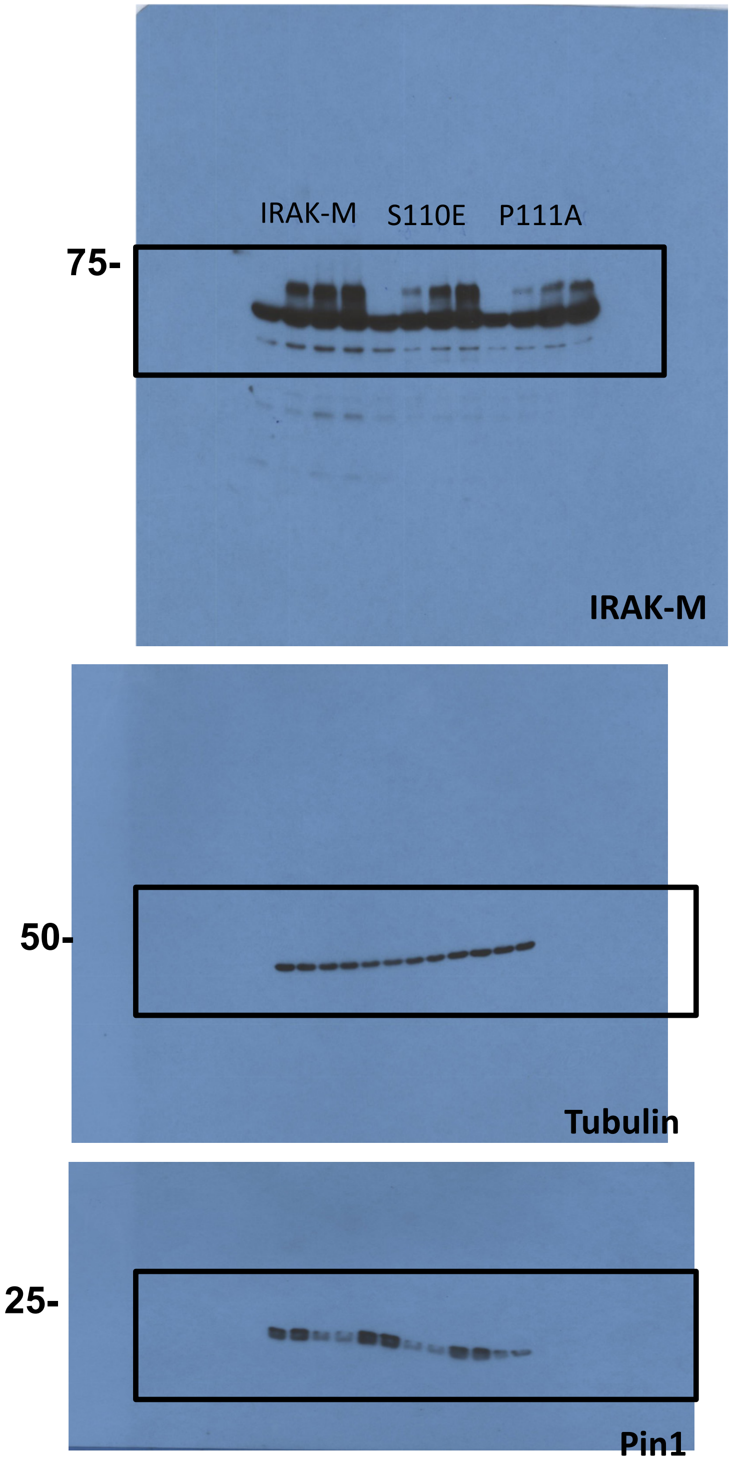

Supplementary Figure 21: Uncropped western blot images for figure 5d. Molecular weight (kDa) of the protein bands are indicated on the left side of the panels. Antibodies used to probe the nitrocellulose membranes are indicated on the bottom right. Rectangles represent the cropped images shown in figures. IRAK-M and its different mutants are indicated on the top of the blot.

Figure 5f

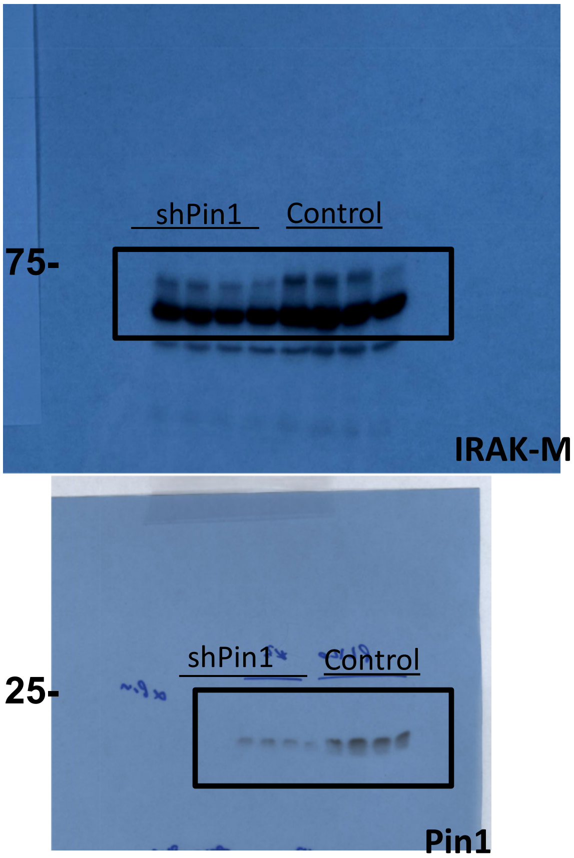

Supplementary Figure 22: Uncropped western blot images for figure 5f. Molecular weight (kDa) of the protein bands are indicated on the left side of the panels. Antibodies used to probe the nitrocellulose membranes are indicated on the bottom right. Rectangles represent the cropped images shown in figures. control and shPin1 extracts are indicated on the top of the blot.

Figure 6e

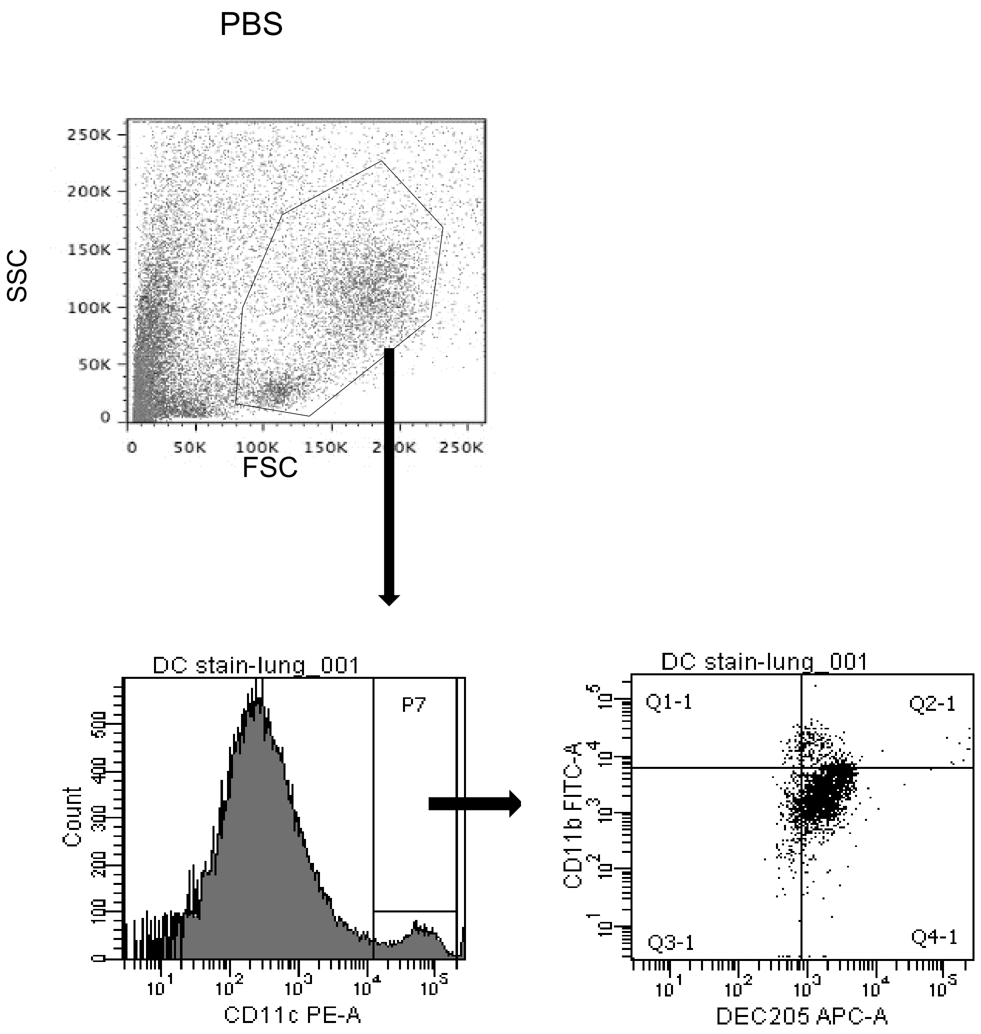

Supplementary Figure 23: FACS analysis and sequential gating for figure 6e. SSC/FSC representative for digested lungs. The gated regions were analyzed for the expression of CD11c⁺ cells. The CD11c⁺ cells were further analyzed for the co expression of DEC205⁺ CD11b⁺ cells. Arrows are indicating the sequential work flow.

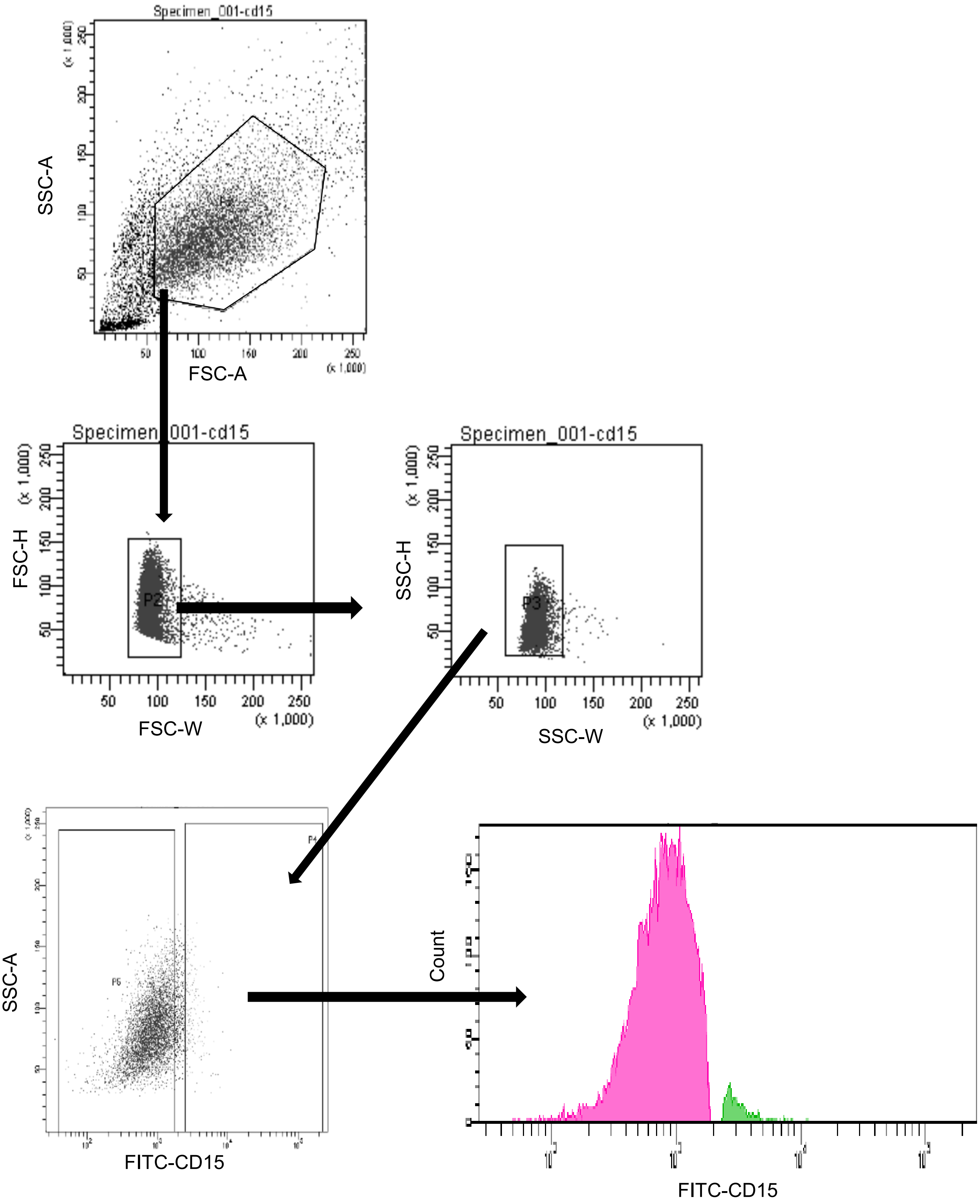

Supplementary Figure 24: FACS analysis and sequential gating for CD15^{+} and cell sorting. SSC/FSC representative of cells from Human patient BAL fluid. The gated regions were analyzed for the expression of CD15^{+}cells and the positive cells were cell sorted. Arrows are indicating the sequential work flow.

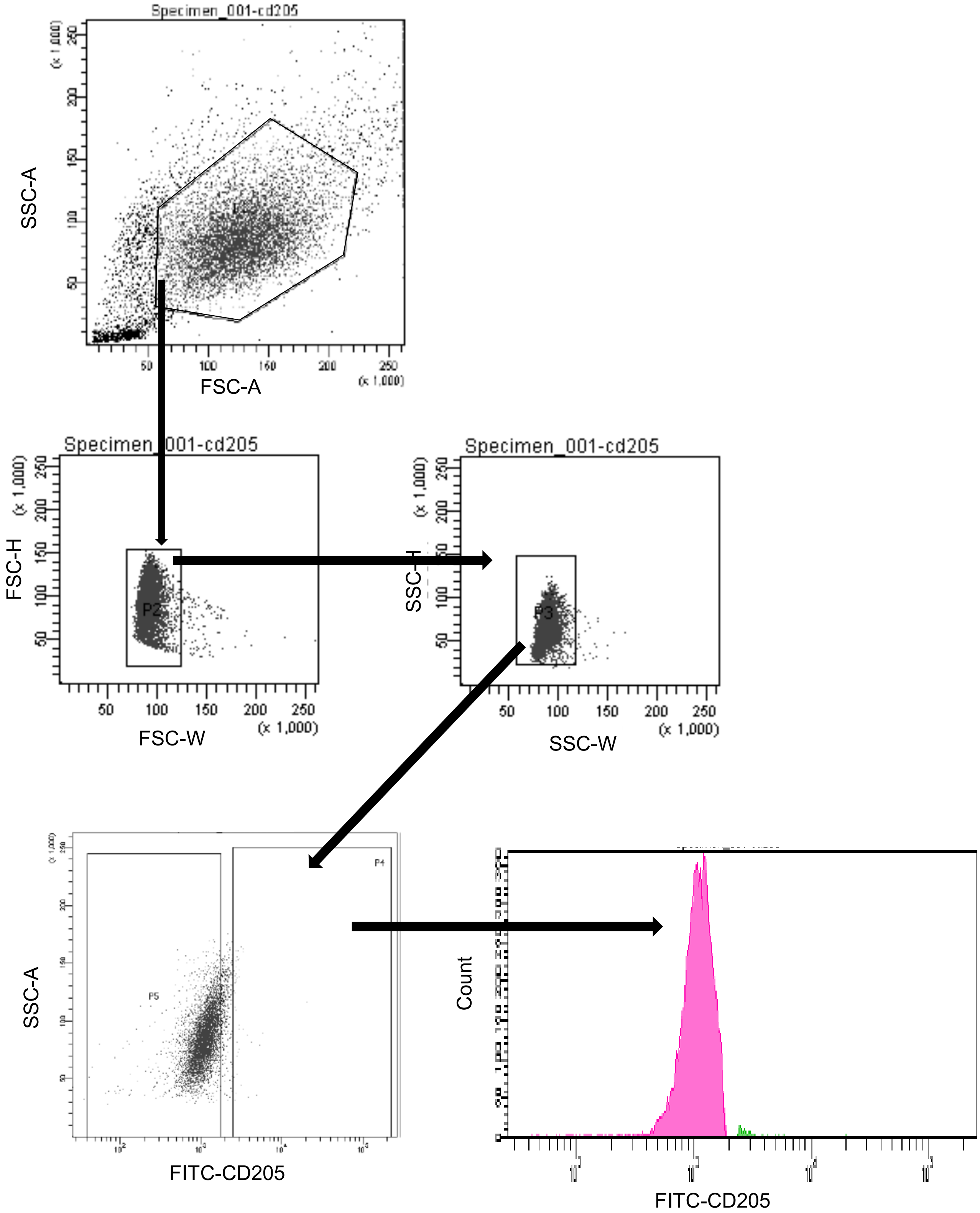

Supplementary Figure 25: FACS analysis and sequential gating for CD205^{+} and cell sorting. SSC/FSC representative of cells from Human patient BAL fluid. The gated regions were analyzed for the expression of CD205^{+}cells and the positive cells were cell sorted. Arrows are indicating the sequential work flow.

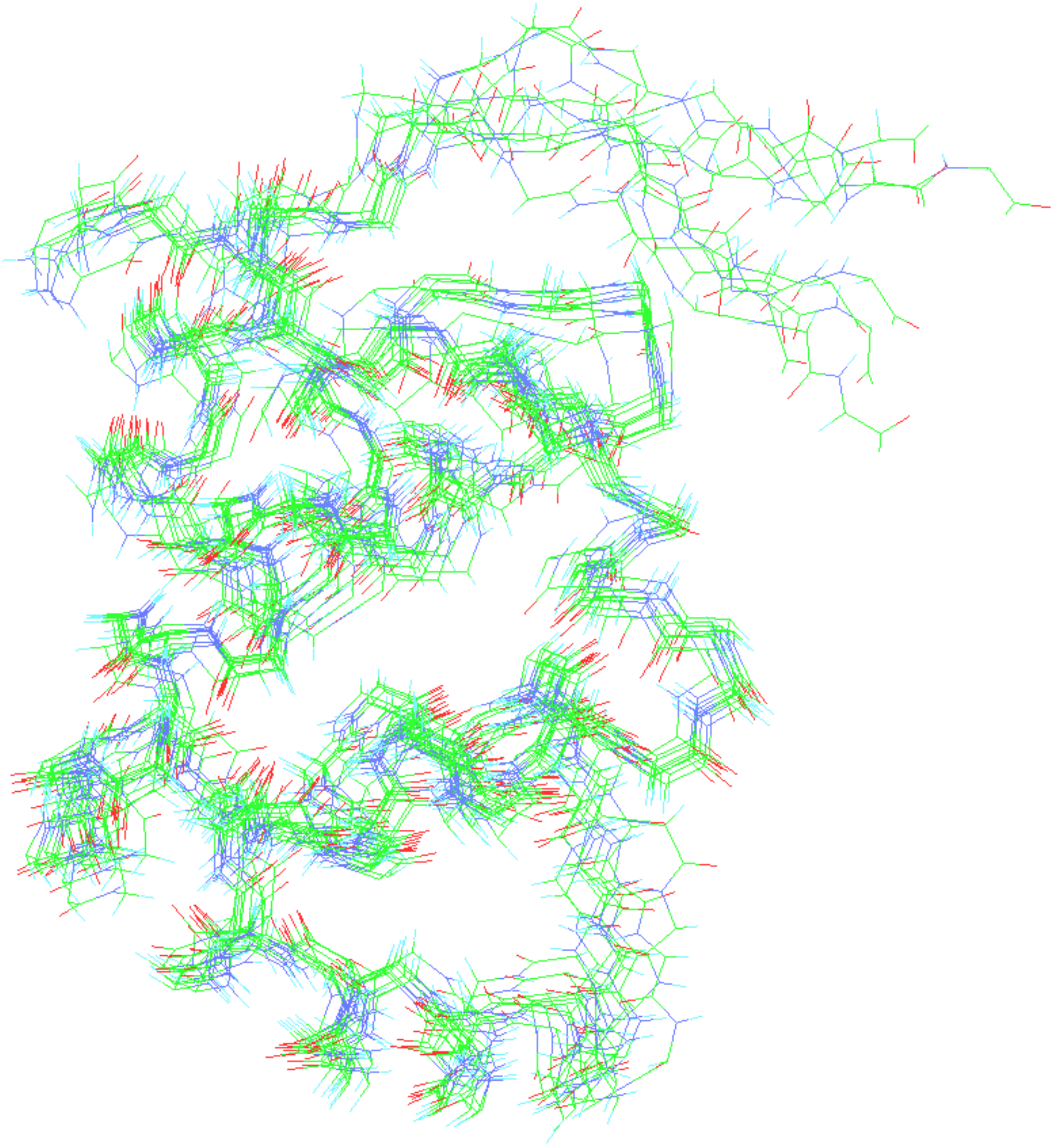

Supplementary Figure 26:

Superposition of ten low energy IRAK-M DD backbone structures from the ensemble of NMR-derived structures (PDB ID 5UKE). Residues A10 through S112 are included in the overlay. The image was generated using SwissPdbView.
